# RB loss in resistant *EGFR* mutant lung adenocarcinomas that transform to small-cell lung cancer

**DOI:** 10.1038/ncomms7377

**Published:** 2015-03-11

**Authors:** Matthew J. Niederst, Lecia V. Sequist, John T. Poirier, Craig H. Mermel, Elizabeth L. Lockerman, Angel R. Garcia, Ryohei Katayama, Carlotta Costa, Kenneth N. Ross, Teresa Moran, Emily Howe, Linnea E. Fulton, Hillary E. Mulvey, Lindsay A. Bernardo, Farhiya Mohamoud, Norikatsu Miyoshi, Paul A. VanderLaan, Daniel B. Costa, Pasi A. Jänne, Darrell R. Borger, Sridhar Ramaswamy, Toshi Shioda, Anthony J. Iafrate, Gad Getz, Charles M. Rudin, Mari Mino-Kenudson, Jeffrey A. Engelman

**Affiliations:** 1 Massachusetts General Hospital Cancer Center, Massachusetts General Hospital, 55 Fruit Street, Boston, Massachusetts 02114, USA; 2 Department of Medicine, Harvard Medical School, 25 Shattuck Street, Boston, Massachusetts 02115, USA; 3 Memorial Sloan Kettering Cancer Center, Thoracic Oncology Service, 1275 York Avenue, New York, New York 10065, USA; 4 Broad Institute of MIT and Harvard, Cancer Genome Comparative Analysis Group, 415 Main Street, Cambridge, Massachusetts 02142, USA; 5 Department of Pathology, Massachusetts General Hospital Cancer Center, 55 Fruit Street, Boston, Massachusetts 02114, USA; 6 Department of Pathology, Harvard Medical School, 25 Shattuck Street, Boston, Massachusetts 02115, USA; 7 Molecular Profiling Laboratory, Massachusetts General Hospital Cancer Center, Massachusetts General Hospital, 55 Fruit Street, Boston, Massachusetts 02114, USA; 8 Department of Pathology, Beth Israel Deaconess Medical Center, Harvard Medical School, 330 Brookline Avenue, Boston, Massachusetts 02115, USA; 9 Department of Medicine, Division of Hematology/Oncology, Beth Israel Deaconess Medical Center, Harvard Medical School, 330 Brookline Avenue, Boston, Massachusetts 02115, USA; 10 Department of Medical Oncology, Belfer Institute of Applied Science, Dana Farber Cancer Institute, 450 Brookline Avenue, Boston, Massachusetts 02215, USA; 11 Department of Medicine, Brigham and Women’s Hospital and Harvard Medical School, 75 Francis Street, Boston, Massachusetts 02115, USA

## Abstract

Tyrosine kinase inhibitors are effective treatments for non-small-cell lung cancers (NSCLCs) with *epidermal growth factor receptor* (*EGFR*) mutations. However, relapse typically occurs after an average of 1 year of continuous treatment. A fundamental histological transformation from NSCLC to small-cell lung cancer (SCLC) is observed in a subset of the resistant cancers, but the molecular changes associated with this transformation remain unknown. Analysis of tumour samples and cell lines derived from resistant *EGFR* mutant patients revealed that Retinoblastoma (RB) is lost in 100% of these SCLC transformed cases, but rarely in those that remain NSCLC. Further, increased neuroendocrine marker and decreased *EGFR* expression as well as greater sensitivity to BCL2 family inhibition are observed in resistant SCLC transformed cancers compared with resistant NSCLCs. Together, these findings suggest that this subset of resistant cancers ultimately adopt many of the molecular and phenotypic characteristics of classical SCLC.

## Abstract (web summary)

Resistance to tyrosine kinase inhibitors occurs in treatments of non-small-cell lung cancers (NSCLCs) with EGFR mutations but the mechanisms underlying this acquired resistance are unknown. Here the authors examine the molecular changes that occur in resistant cancers that transition from NSCLC to small-cell lung cancer phenotype and implicate loss of retinoblastoma in this process.

## Graphical abstract

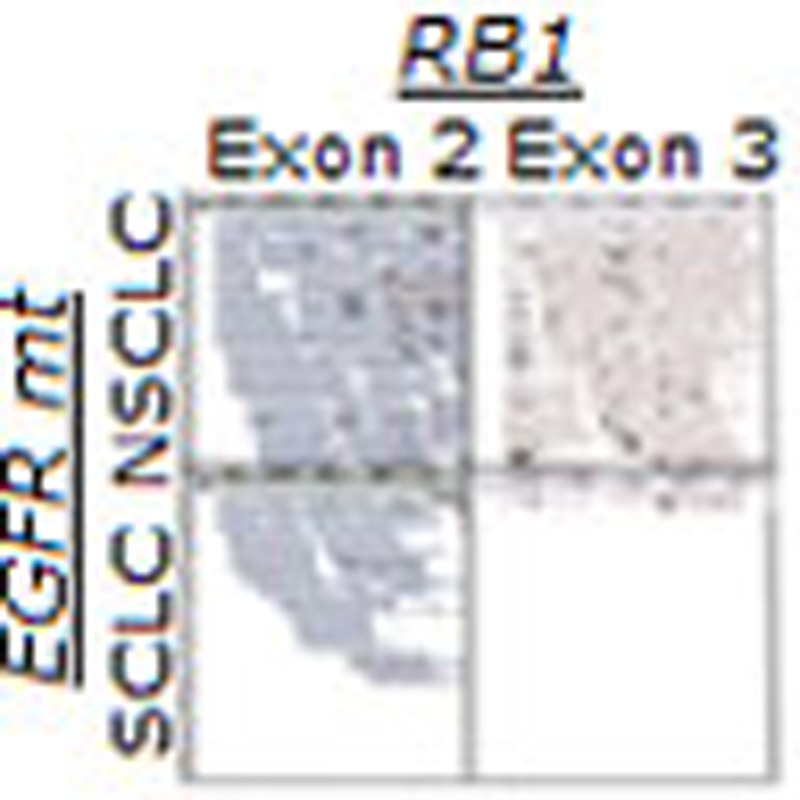

The tyrosine kinase inhibitors (TKIs) gefitinib, erlotinib and afatinib are effective therapies for non-small-cell lung cancers (NSCLCs) harbouring activating mutations in the *epidermal growth factor receptor* (*EGFR*). The majority of these patients achieve robust responses, with marked tumour shrinkage, abatement of symptoms and improved outcome compared with chemotherapy^1^,^2^,^3^,^4^,^5^. Despite initial efficacy, resistance to TKIs invariably develops, with disease progression after an average of approximately 12 months^6^. The implementation of repeat biopsy programmes at the time of clinically apparent resistance has been instrumental to the understanding of the molecular mechanisms underlying acquired resistance to EGFR TKIs. We previously reported the results of a cohort of patients undergoing repeat biopsy in which we identified secondary mutations in *EGFR* (T790M), amplification of the *MET* receptor tyrosine kinase and mutations in *PIK3CA*, all of which confer resistance to TKI via reactivation of key downstream signalling pathways^7^. In addition, a subset of resistant tumours underwent phenotypic/histological changes, namely transformation to small-cell lung cancer (SCLC) and epithelial-to-mesenchymal transition. Importantly, the tumours that transformed to SCLC harboured the original activating *EGFR* mutation, suggesting direct evolution from the initial cancer, rather than a distinct, second primary cancer. The phenomenon of SCLC transformation in resistant *EGFR* mutant cancers had been previously identified in individual patient case reports^8^,^9^,^10^,^11^,^12^ and has subsequently been confirmed in another repeat biopsy patient cohort^13^. However, the molecular details underlying this histological change and resistance to EGFR TKI therapy, as well as the relatedness of *EGFR* mutant SCLC to classical SCLC, remain unclear. Here, we characterize the molecular changes that occur in NSCLC to SCLC transformed TKI-resistant *EGFR* mutant cancers. Our results indicate that SCLC transformed resistant cancers take on many features of classical SCLC, including universal alterations to the *RB* tumour suppressor, gene expression profiles similar to classical SCLC, which include reduced or absent EGFR expression, and heightened sensitivity to BCL-2 family inhibition.

## Results

### Transformed SCLC RNA profiles mimic classical SCLC

**Figure 1:**
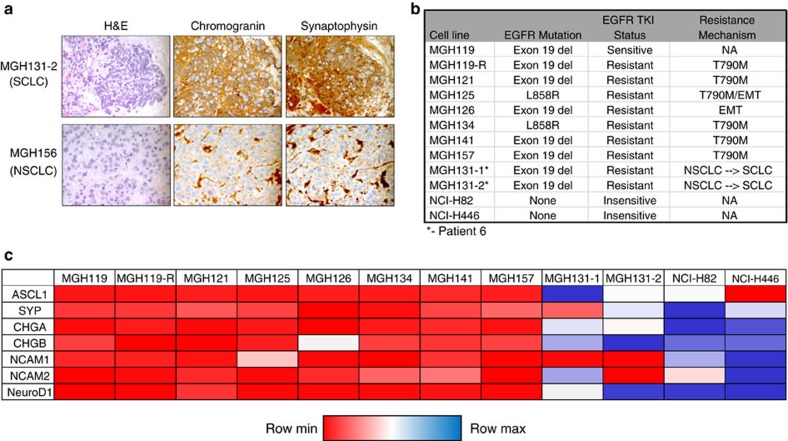
SCLC transformed cell lines exhibit neuroendocrine (NE) features. (**a**) Haematoxylin and eosin (H&E) staining and IHC for NE markers chromogranin and synaptophysin were performed on xenografts derived from *EGFR* mutant MGH131-2 SCLC and MGH156 NSCLC cells. (**b**) *EGFR* mutation status, TKI sensitivity and resistance mechanism for the patient-derived cell lines analysed in **c**. (**c**) Gene expression array data of NE marker expression across a panel of cell lines derived from TKI-resistant patients (*n*=10). NCI-H82 and NCI-H446 are classical SCLC cell lines used as controls for NE marker expression. Red indicates lower expression and blue indicates higher expression.

To perform these analyses, we amassed a collection of 11 *EGFR* mutant cancer samples (from nine patients) that underwent transformation to SCLC at the time of acquired resistance to EGFR TKI therapy under the auspices of an institutional review board (IRB)-approved protocol (Supplementary Table 1). As reported previously, all of the resistant SCLC cancers harboured the original activating *EGFR* mutation^7^. Cell lines derived from resistant patient biopsies have been valuable tools to study acquired resistance to TKIs in lung cancer^14^,^15^,^16^, and two such models (MGH131-1 and MGH131-2) were derived from two different biopsies (taken several months apart) of an erlotinib-resistant patient whose cancer had transformed to SCLC (Patient #6, Supplementary Table 1). Before erlotinib, this patient’s cancer had NSCLC histology. As expected, these biopsy-derived cell lines continue to harbour the *EGFR* exon 19 deletion mutation in a majority of *EGFR* alleles (variant allele frequency ~60% for both cell lines) indicating that most, if not all, of the cells are *EGFR* mutation positive. Histological analyses of xenograft tumours derived from these cell lines confirmed SCLC histology and expression of neuroendocrine (NE) markers in contrast to xenograft tumours derived from a resistant *EGFR* mutant cancer that maintained NSCLC histology (Fig. 1a). Hierarchical clustering analysis of RNA expression revealed that the two cell lines derived from a resistant *EGFR* mutant SCLC more closely resembled classical SCLC cell lines (including expression of NE markers) than cell lines derived from resistant *EGFR* mutant NSCLCs (Fig. 1b,c and Supplementary Fig. 1a,b). In addition, we profiled the expression of ten microRNAs (miRNAs) that had been previously identified to be the most differentially regulated between adenocarcinoma and SCLC cell lines^17^. The expression pattern of both the MGH131-1 and MGH131-2 cell lines more closely resembled classical SCLCs (Supplementary Fig. 1c). Notably, the MGH131-1 cells expressed miRNA that were also expressed in NSCLC. The MGH131-1 cells more closely resemble the mesenchymal subtype of SCLC described by Berns and colleagues (E-cadherin low, Vimentin high, less positive for NE markers, more adherent growth in culture)^18^ than the MGH131-2 cells (Supplementary Fig. 1d). However, altogether, these findings reveal that the EGFR mutant SCLC transformed cells resemble classical SCLC with respect to mRNA and miRNA expression.

### Resistant transformed SCLCs lose EGFR expression

**Figure 2:**
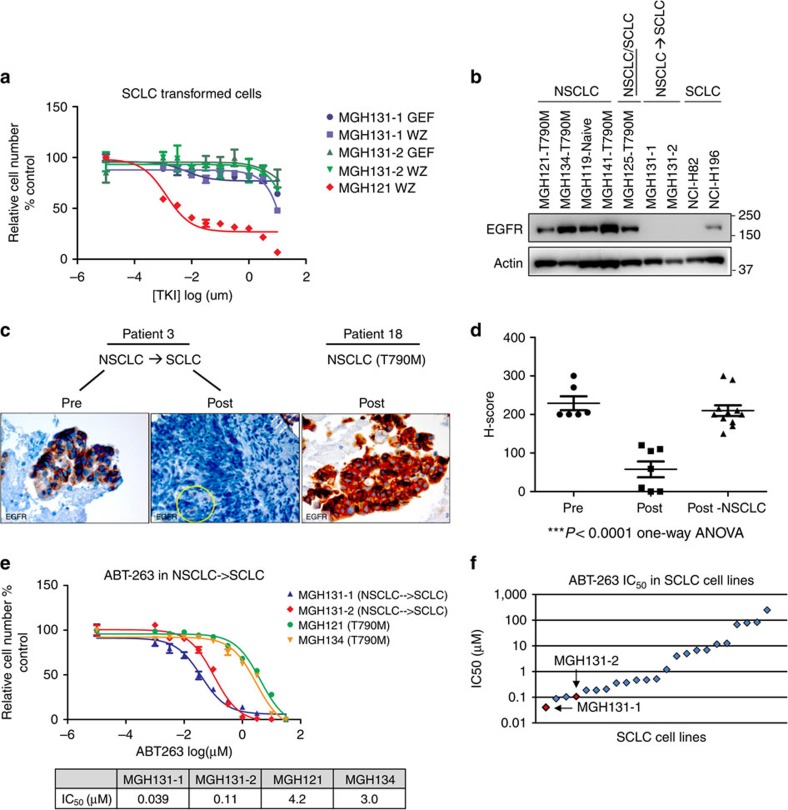
Resistant SCLCs respond to ABT-263 and lose EGFR expression. (**a**) The resistant *EGFR* mutant SCLC cell lines MGH131-1 and MGH131-2, and a resistant *EGFR* mutant NSCLC cell line that harbour T790M, MGH121, were treated with indicated concentrations of Gefitinib (GEF) or the third-generation EGFR inhibitor WZ4002 (WZ) for 72 h. Cell viability was measured with the CellTiter-Glo assay. Experiments were performed in quadruplicate and error bars depict the standard error of the mean for each data point. (**b**) Representative blot of lysates from a panel of patient-derived resistant *EGFR* mutant cell lines and classical SCLC cell lines was probed with antibodies specific to total EGFR and actin (MGH119 was derived from a TKI naïve patient). Lysates from this panel were also probed in Supplementary Fig. 1c. (**c**) IHC staining for total EGFR on a representative pair of matched pre- and post-resistant samples from a patient whose resistant *EGFR* mutant cancer transformed from NSCLC to SCLC (Patient #3, left and middle) and a resistant *EGFR* mutant cancer that remained NSCLC (patient #18, right). The yellow circle indicates EGFR-positive endothelial cells in the resistant *EGFR* mutant SCLC. (**d**) Quantification (H-score) of EGFR staining from pair-matched pre (*n*=6) and post-resistant (*n*=7) samples from cancers that transformed into SCLC upon the development of resistance. Resistant *EGFR* mutant cancers that maintained NSCLC histology are shown for comparison (*n*=11). ***P<0.0001 one-way analysis of variance (ANOVA) with Bonferroni *post-hoc* test. (**e**) Patient-derived TKI-resistant cell lines from resistant SCLC (MGH131-1 and MGH131-2), and T790M-positive NSCLC (MGH121 and MGH134) were treated with indicated concentrations of ABT-263 for 72 h and cell viability was measured with the CellTiter-Glo assay. Each data point was repeated in quadruplicate and error bars represent the standard error of the mean. Bottom—IC_50_ values for ABT-263 for each cell line. (**f**) ABT-263 IC_50_ values compared with those from a panel of SCLC cell lines^37^.

We next tested the MGH131-1 and MGH131-2 cells for their sensitivity to EGFR TKIs. Cell viability assays indicated that both SCLC transformed cell lines were highly resistant to gefitinib as well as the third-generation EGFR inhibitor, WZ4002, which effectively inhibits both activating mutations and the T790M resistance mutation (Fig. 2a)^19^. In contrast, a patient-derived resistant cell line that retained NSCLC histology and had a T790M mutation (MGH121) was exquisitely sensitive to WZ4002 (Fig. 2a). Thus, the *EGFR* mutant SCLC cell lines retain resistance to EGFR inhibition, similar to what is observed clinically.

To understand why SCLC transformed cells are insensitive to EGFR TKIs despite continued presence of the *EGFR* activating mutation, we measured the levels of EGFR to determine if transformation to SCLC had resulted in altered expression. Western blotting revealed an absence of EGFR expression specifically in the *EGFR* mutant SCLC transformed cell lines (Fig. 2b). To determine whether EGFR expression is commonly lost in *EGFR* mutant lung cancers that transform to SCLC, we performed IHC analysis on seven resistant cases of *EGFR* mutant cancers that had transformed to SCLC along with ten cases that retained NSCLC histology. As shown in Fig. 2c,d, there was a marked decrease in EGFR expression in the SCLC resistant tumours compared with baseline, but EGFR expression was intact in resistant *EGFR* mutant NSCLCs. Indeed, interrogation of the expression data from the cancer cell line encyclopedia^20^ (CCLE) database revealed that classical SCLC cell lines have significantly reduced levels of *EGFR* mRNA compared with adenocarcinoma cell lines (Supplementary Fig. 2a). Similarly, SCLC transformed *EGFR* mutant-resistant cell lines had lower levels of *EGFR* mRNA compared with NSCLC-resistant models (Supplementary Fig. 2b). These data suggest that SCLC transformed *EGFR* mutant cancers lose expression of EGFR, as is typical of classical SCLC, and thus it is not surprising that they are no longer sensitive to EGFR inhibition.

### SCLC transformed cell lines are sensitive to ABT-263

The BCL-2, BCL-XL inhibitor, ABT-263, is one of the few therapies to date to exhibit marked efficacy against SCLC in laboratory studies^21^, and although recent results from clinical trials with single-agent ABT-263 demonstrated responses in only a minority of SCLC patients^22^, combinations with this agent are being explored^23^. SCLC transformed *EGFR* mutant cells were highly sensitive to single-agent ABT-263 and markedly more sensitive than EGFR-TKI-resistant NSCLC cell lines harbouring the T790M resistance mutation (Fig. 2e). ABT-263 treatment induced a robust apoptotic response in *EGFR* mutant SCLC compared with the resistant *EGFR* mutant NSCLC (Supplementary Fig. 2c). We next compared the IC_50_ values of ABT-263 in the SCLC transformed cell lines to a panel of 21 classical SCLC cell lines, and found that MGH131-1 and MGH131-2 were among the most sensitive to ABT-263 (Fig. 2f). Indeed, ABT-263 was significantly more active than gefitinib in MGH131-1 and MGH131-2 cells (Supplementary Fig. 2d). These results underscore the potential of ABT-263 as part of combination strategy to treat *EGFR* mutant patients with NSCLC to SCLC transformation. In total, the gene expression and drug sensitivity of the SCLC transformed cells more closely resembles classical SCLC than *EGFR* mutant NSCLC. These data are further supported by the clinical observations that *EGFR* mutant SCLCs are highly sensitive to SCLC chemotherapy regimens^7^.

### DNA sequencing reveals genetic lesions specific to resistant SCLC

**Figure 3:**
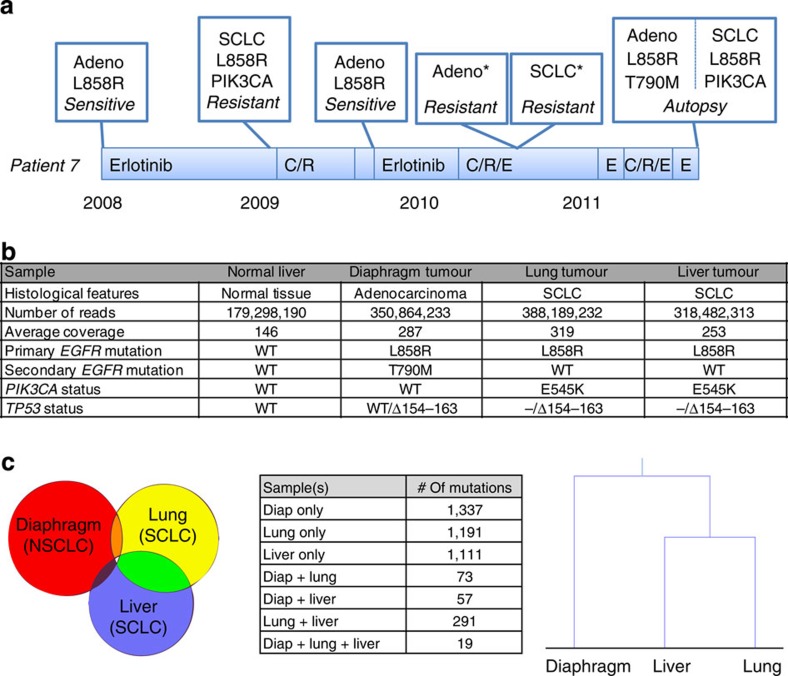
NGS reveals specific genetic alterations in resistant SCLCs. (**a**) Treatment and biopsy history of Patient #7. Treatment regimens and findings from sample collection are noted. C/R, chemotherapy+radiation; E, Erlotinib. *Adeno and SCLC components were from a pleural effusion and bone biopsy, respectively. (**b**) Histological features, sequencing statistics and genotypes of the samples analysed by exome sequencing. (**c**) Left, Venn diagram depicting the unique and shared mutations across the three resistant tumours. Center, Number of unique and shared mutations across the three samples. Right, Inferred clonal evolution of the three resistant tumours based on number of shared and unique mutations.

In our previous report^7^, we described a patient (Patient #7) who had been biopsied multiple times over the course of their disease. In this patient, both *EGFR* mutant adenocarcinoma and SCLC had been observed at different times. This patient ultimately passed away, and at autopsy, both SCLC and NSCLC were identified (Fig. 3a). The oscillating pattern of adenocarcinoma and SCLC that was observed suggested that different clones were selected depending on the selective pressure of the applied treatment (conceptual schematic shown in Supplementary Fig. 3a). The autopsy that was performed identified two SCLC transformed tumours (one each from the liver and lung) as well as a diaphragmatic tumour that retained adenocarcinoma histology (Supplementary Fig. 3b). All three lesions contained the original activating *EGFR* mutation, and the diaphragmatic NSCLC tumour harboured the *EGFR* resistance mutation, T790M, whereas the SCLCs did not (Fig. 3b). All samples (along with normal liver tissue) were analysed by WES. The variant allele frequencies of the activating *EGFR* mutation were 66% and 77% in the resistant SCLC samples, consistent with earlier results demonstrating that the *EGFR* mutation is harboured in the transformed SCLC cells^7^. Clonal analyses revealed that the two SCLC samples had a greater number of mutations in common with each other (*n*=291) than either shared with the resistant tumour that maintained NSCLC morphology ((*n*=73) shared mutations between the SCLC lung and NSCLC diaphragm, and (*n*=57) shared mutations between the SCLC liver and NSCLC diaphragm; Fig. 3c). This suggests that the two SCLC resistant lesions are more closely related and likely diverged later in the evolution of the resistant disease compared with the adenocarcinoma lesion.

By comparing the genomic variants from these four samples, we were able to look for somatic changes frequently detected in NSCLC and SCLC genomes. Both SCLC transformed samples harboured an activating mutation in *PIK3CA*, which we previously observed in SCLC transformed cases^7^ as well as loss of heterozygosity and an inactivating mutation of *TP53*, which is universally altered in classical SCLC^24^,^25^ (Fig. 3b). In addition, there was a near absence of reads for a portion of the *RB1* gene (<10% of the reads compared with the normal liver and adenocarcinoma), in both resistant SCLC transformed tumours, but not in the resistant adenocarcinoma sample (Supplementary Fig. 3c). This suggests bi-allelic loss of *RB1* specifically in the SCLC transformed tumours. This was particularly noteworthy as RB is invariably lost in classical SCLC^24^,^25^,^26^. Alterations to other frequently altered genes in SCLC such as *MYC*, *PTEN* and *FGFR1* were not detected. Thus, analogous to classical SCLC, alterations to *TP53* and *RB1* were observed in *EGFR* mutant NSCLC to SCLC transformed tumours.

**Figure 4:**
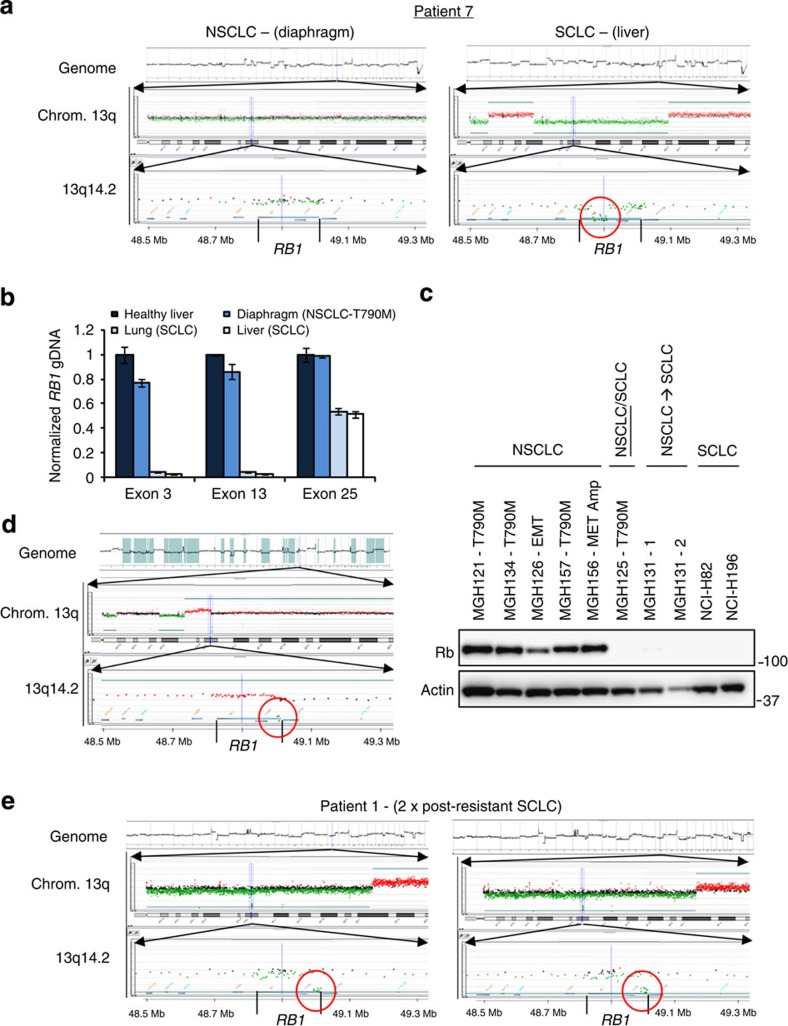
Resistant *EGFR* mutant SCLCs have genetic loss of *RB1*. (**a**) CGH array profiles of a resistant NSCLC tumour (left) and SCLC transformed tumour (right) from Patient #7 at the level of the whole genome (top), chromosome 13q12.12-q32.2 (middle) and the 0.8 Mb region flanking the *RB1* gene (bottom). The *RB1* gene locus is depicted and regions of bi-allelic loss are circled. (**b**) qPCR analysis of *RB1* exons 3, 13 and 25 amplified from genomic DNA from the indicated autopsy specimens from Patient #7. Reactions were carried out in triplicate and error bars representing standard error of the mean are shown. (**c**) Representative blot of lysates from resistant *EGFR* mutant cell lines derived from resistant biopsies along with classical SCLCs was probed with antibodies specific to RB and actin. (**d**) CGH array profile of the MGH131-1 cell line of the whole genome (top), chromosome 13q12.12-q32.2 (middle) and the 0.8 Mb region flanking the *RB1* gene (bottom). The *RB1* gene locus is depicted and regions of bi-allelic loss are circled. (**e**) CGH array profiles of two resistant *EGFR* mutant SCLCs from Patient #1 with depiction of whole genome (top), chromosome 13q12.12-q32.2 (middle) and the 0.8 Mb region flanking the *RB1* gene (bottom). The *RB1* gene locus is depicted and regions of bi-allelic loss are circled.

In the liver SCLC tumour, comparative genomic hybridization (CGH) array analysis revealed that there was a relatively large deletion in one copy of *RB1* that encompassed the entire gene and the surrounding region. This was accompanied by a focal deletion in the second copy that spanned only the middle exons of *RB1* but spared the beginning and end of the gene (Fig. 4a). These deletions were not observed in the resistant cancer with a T790M mutation and NSCLC histology. These results were confirmed by quantitative PCR (qPCR) of different exons of *RB1*, which also demonstrated similar focal loss of *RB1* in the lung SCLC (Fig. 4b).

### RB is universally lost in resistant SCLC patients

The cell lines established from biopsies of resistant *EGFR* mutant lung cancers were assessed for RB expression. Western blotting revealed loss of RB expression specifically in resistant *EGFR* mutant cell lines with SCLC histology (Fig. 4c). Notably, the MGH125 cell line (patient #8) also lacks RB expression. This cell line was generated from a pleural effusion, which demonstrated NSCLC histology, however, a previous liver biopsy of this patient’s cancer revealed a metastatic lesion that had transformed to SCLC (Supplementary Fig. 4a). Thus, this cancer was particularly prone to SCLC transformation. Array CGH analysis revealed a focal deletion of both copies of *RB1* in the MGH131-1 SCLC cell line (Fig. 4d). However, only one copy of *RB1* was lost in the MGH125 cells (Supplementary Fig. 4b). Sequencing of *RB1* from MGH125 cells revealed that the intact copy of *RB1* harboured a nonsense mutation (R445*, Supplementary Fig. 4c), explaining the absence of RB protein expression in these cells (Fig. 4c). Thus, cell lines derived from cancers that either have transformed into SCLC or derived from tumours prone to transform into SCLC both demonstrated genetic loss of *RB1.*

To expand these analyses, we examined the collection of 10 *EGFR* mutant cancer samples (from 9 patients) that underwent transformation to SCLC at the time of acquired resistance as well as the 11 resistant controls that had maintained NSCLC histology (Supplementary Table 1). In one of the SCLC transformed cases, Patient #1, we had sufficient sample from two resistant lesions to harvest DNA and assess the *RB1* locus by array CGH. Concordant with the findings from Patient #7, there was a bi-allelic loss with one relatively large deletion and a second highly focal deletion in both resistant SCLC samples (Fig. 4e).

**Figure 5:**
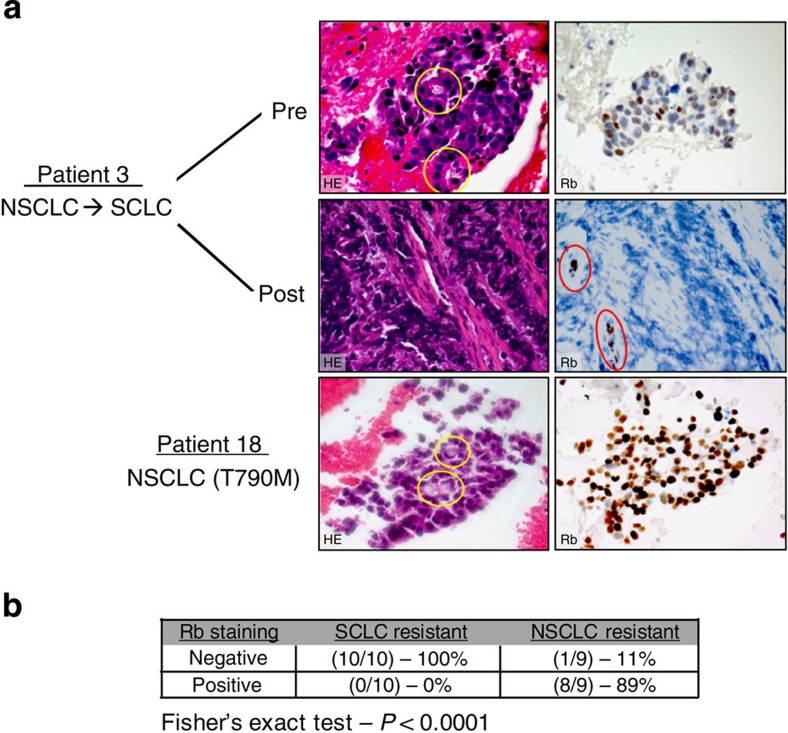
RB is invariably absent in resistant *EGFR* mutant SCLCs. (**a**) Haematoxylin and eosin (HE) staining (left) and the corresponding RB IHC (right) for a representative matched pair of pre-treatment *EGFR* mutant NSCLC and the corresponding post-resistant *EGFR* mutant SCLC (Patient #3, top, middle). A resistant *EGFR* mutant cancer that maintained adenocarcinoma histology and acquired a T790M *EGFR* mutation is shown for comparison (Patient #18, bottom). Yellow circles indicate gland formation of the moderately differentiated adenocarcinomas. Red circles indicate positive staining in endothelial cells. (**b**) Results of RB IHC staining of EGFR mutant-resistant cancers that underwent the transformation from NSCLC to SCLC (SCLC Resistant, *n*=10) and those that retained an adenocarcinoma histology (NSCLC Resistant, *n*=9). Resistant *EGFR* mutant SCLC is significantly more likely than resistant *EGFR* mutant NSCLC to have loss of RB expression (*P*<0.0001, Fisher’s exact test).

Because we did not have sufficient tissue from the remaining samples to perform genetic analyses, we developed an immunohistochemistry (IHC) assay to examine RB expression in the larger cohort of *EGFR* mutant, SCLC transformed samples. IHC has some potential advantages for determining RB status: (i) IHC requires minimal tumour material, which is a common obstacle in these clinical samples, (ii) RB deficiency is detected even when there is loss due to mechanisms other than bi-allelic deletion, such as nonsense mutations and (iii) direct visualization of individual cells allows precise interpretation in cases that contain a large proportion of stroma, which may confound next-generation sequencing (NGS) and CGH array analyses. Control experiments confirmed the robustness of the IHC assay. For example, it accurately detected strong expression in RB-positive tumours, weak RB expression in tumours with reduced levels mediated by short hairpin RNA (shRNA) knockdown and an absence of RB in tumours with dual copy loss (Supplementary Fig. 5). IHC analyses were completed on ten resistant *EGFR* mutant SCLC samples and revealed complete loss of RB expression in all cases (Fig. 5a,b and Table 1). As a control, RB IHC was performed on the 11 resistant tumours that remained NSCLC. RB expression was intact in all but one sample. These data reveal selective loss of RB expression in *EGFR* mutant lung cancers that transform to SCLC upon the development of resistance (*P*<.0001, Fisher’s exact test). Thus, *EGFR* mutant lung cancers that transform to SCLC invariably lose RB expression, similar to classical SCLC. In total, these findings suggest that chronic EGFR inhibition in *EGFR* mutant lung adenocarcinomas can lead to the development of cancers that adopt the genetic, histologic, expression and drug sensitivity profiles of classical SCLC.

The universal nature of the RB loss is suggestive that this may be a necessary event for the SCLC-resistant tumours to emerge. Although RB is lost in classical SCLC, it is not known if RB loss is necessary for NE differentiation or the growth and survival of cells that have differentiated along a NE lineage. It is notable that shRNA-mediated depletion of RB in gefitinib-sensitive NSCLC cell lines did not alter the sensitivity to gefitinib (Supplementary Fig. 6a). Furthermore, generating TKI-resistance *in-vitro* or *in-vivo* in *EGFR* mutant cancer cell lines engineered to have loss of RB expression did not yield resistant cells/tumours with acquisition of NE marker expression or SCLC morphology (Supplementary Fig. 6b,c). These results suggest that loss of RB is likely necessary in order for acquired resistance via transformation to SCLC to develop, but it is not sufficient on its own to promote it. The latter point is further supported by our discovery of a few examples of RB-deficient adenocarcinomas. Indeed, two erlotinib-resistant cell lines (MGH125 and MGH141), a resistant patient sample (Patient #10) and two out of four pre-treatment adenocarcinoma samples from patients whose cancers transformed to SCLC (Patients #2 and #6), were also negative for RB. Although rare, the existence of these RB-deficient adenocarcinomas serves as further evidence that loss of RB alone is insufficient to promote transformation to SCLC.

## Discussion

Acquired resistance is a major problem limiting the clinical efficacy of targeted therapies. Repeat biopsy studies have led to the identification of the resistance mechanism in a majority of *EGFR* mutant NSCLC patients that have progressed on EGFR TKIs^7^,^13^. One unexpected finding from these studies was the discovery that 5–15% of patient tumours undergo transformation to SCLC histology upon acquisition of resistance. From a historic perspective of lung cancer classification, this observation was a surprise, as differentiation into a NSCLC- or SCLC-type cancer was thought to occur early in tumorigenesis. Furthermore, the typical presentation of these diseases were quite distinct, with *EGFR*-mutant adenocarcinoma occurring primarily in never-smokers and displaying a more indolent natural history compared with classical SCLC, which occurs almost exclusively in heavy smokers and tends to metastasize early and grow rapidly. Indeed, the SCLC transition seen in *EGFR*-mutant patients is often accompanied by a change in the clinical behaviour of the disease, with rapid acceleration in the growth rate, initial responsiveness to chemotherapy followed by rapid clinical deterioration^7^. However, repeat biopsy studies have consistently suggested that the SCLC transformed cancers represent an evolution from the initial adenocarcinomas rather than a second coincident cancer, because the activating driver *EGFR* mutations are identical to the original adenocarcinomas in all cases. To date, the mechanistic details regarding this transition are unknown. This study revealed genetic changes specifically associated with the transformation to SCLC, provided insight into why these tumours are no longer sensitive to EGFR TKIs, and determined a potential therapeutic that could be incorporated into future treatment strategies for this subset of resistant cancers.

Assessment of RB status by a combination of next-generation sequencing, array CGH, qPCR and IHC analyses revealed that RB was lost in 11 out of 11 SCLC transformed samples, a result that mirrors classical SCLC, in which RB is altered in an overwhelming majority of cases^24^,^25^,^26^. Interestingly, RB knockdown experiments in *EGFR* mutant cell lines suggest that RB loss was insufficient to cause resistance or induce NE differentiation. It is notable that these knockdown studies were performed in established *EGFR* mutant cell lines. Such cell lines may not possess the pluripotent cells that are present in a tumour *in vivo* that may have the capacity to differentiate along different lineages including SCLC. We speculate that in these pluripotent cells that differentiation to NSCLC is favoured when EGFR is active, as EGFR activity has been associated with promoting alveolar differentiation^27^ (Supplementary Fig. 7, left panel). Conversely, following treatment with EGFR-TKI, the resistant pluripotent cells, which may have accumulated additional genetic alterations (such as loss of *RB1* and *TP53*) and maintain a different epigenetic state, are able to differentiate and subsequently expand along a lineage (SCLC) that does not require EGFR signalling (Supplementary Fig. 7, right panel). It is also interesting to note that the absence of EGFR signalling induced by the TKI may remove the impetus to differentiate along the NSCLC lineage, thereby facilitating differentiation along the other lineage. Along these lines, there have been case reports of treatment naïve *EGFR* mutant SCLC^12^,^28^, reinforcing the notion that the cell of origin of *EGFR* mutant lung cancers may have the potential to differentiate along a NE lineage. Notably, we assessed one such case (Patient #19, Table 1), and this cancer had loss of RB and EGFR expression, similar to the cases of *EGFR* mutant SCLC observed in the setting of acquired resistance to EGFR TKI.

We cannot rule out that *EGFR* mutant SCLC pre-existed before treatment with the EGFR TKI. We have carefully reviewed the histology of these samples and we do not observe a mix of NSCLC and SCLC histology in the pre-treatment tumours. Of course, this does not rule out the possibility that a very small percentage of SCLC cells that are below our detection limit do pre-exist (especially, as the biopsies only sample a minute fraction of the patients’ total cancer burden). However, from a clinical perspective, we feel that it is unlikely that these SCLCs were present from the onset of the disease in a majority of these cases because when the SCLC surfaces in the clinic, it progresses quite rapidly (like classical SCLC)^7^. In many of these cases, the TKI-induced remissions last for years and then suddenly the patient develops explosive SCLC. It seems unlikely (but, not impossible) that the same explosive cancer was present for all of those years while the patients were in remission. In such cases, we favour a model in which the cells that survived treatment undergo further ‘evolution’ to become the *bona fide* SCLC that ultimately presents in the clinic (as described above and shown in Supplementary Fig. 7).

The finding that all *EGFR* mutant SCLC transformed samples have low/absent EGFR expression compared with pre-resistant controls provides insight into the explanations for the lack of sensitivity of these cancers to TKI. We speculate that, upon transformation to SCLC, they take on many of the characteristics of classical SCLC, which normally do not express EGFR or rely on its activity for growth and survival^29^. Thus, the treatment strategies that will provide the most benefit to this subset of cancers will likely resemble those that are most effective for classical SCLC.

Our data reveal that *EGFR* mutant cancers that transform to SCLC also undergo significant epigenetic changes. Hierarchical clustering analysis of RNA expression data demonstrated that cell lines derived from SCLC transformed resistant biopsies share gene expression profiles more closely related to classical SCLC cell lines than other TKI-resistant cell lines that maintained NSCLC histology. Similarly, miRNA analyses revealed that SCLC transformed cells express miRNAs that are commonly upregulated in classical SCLC. It is notable, however, that the SCLC transformed cells also express a subset of miRNAs that are typically expressed in adenocarcinoma but not SCLC. Furthermore, DNA methylation analysis of resistant SCLC tumours from patient # 7 revealed a methylation pattern more consistent with adenocarcinoma than SCLC (Supplementary Fig. 8). These results indicate that SCLC transformed cancers may retain some features consistent with their adenocarcinoma origins. Importantly, however, from a genetic, mRNA expression profile, and clinical perspective these cancers behave like classical SCLC.

In summary, this study reveals some of the key molecular changes associated with *EGFR* mutant lung adenocarcinomas that transform to SCLC upon acquisition of resistance to EGFR TKI. As novel therapeutic approaches that inhibit EGFR more efficiently become widely implemented^30^,^31^,^32^, we speculate that the relative frequency of NSCLC to SCLC transformation in the setting of acquired resistance may increase moving forward, further underscoring the importance of understanding the basis for this transformation as well as treatment strategies to overcome it.

## Methods

### Patients

EGFR mutant NSCLC patients underwent biopsies before and after acquiring resistance to erlotinib therapy as per standard clinical care over an 8-year period from 2005 to 2013. Standard histology and the SNaPshot assay were carried out to determine histological subtype and genotype of each sample^33^. An IRB-approved protocol was followed to review the electronic medical record for relevant clinical information. Patient-derived cell lines were generated under an IRB-approved protocol, which required prospective informed consent from participating patients.

### Reagents and cell culture

PC9, HCC827, MGH119, MGH119-R, MGH121, MGH134, MGH141, MGH157, NCI-H446, NCI-H196 and NCI-H82 cells were cultured in RPMI 1640 supplemented with 10% fetal bovine serum. MGH125, MGH126, MGH131-1 and MGH131-2 cells were cultured in ACL4 supplemented with 10% fetal bovine serum. NCI-H446, NCI-H196 and NCI-H82 cells were obtained from the Center for Molecular Therapeutics at MGH. PC9 and HCC827 cells were a gift from Pasi Jänne. Gefitinib and WZ4002 were purchased from Selleck, Abt-263 was purchased from Active Biochem. All compounds were reconstituted in dimethylsulphoxide for cell culture experiments. Antibodies for RB, actin, NCAM, synaptophysin, NeuroD, pAkt T308, pERK T202/Y204 and total Akt were purchased from Cell Signaling Technology. pEGFR Y1068 and chromogranin A were purchased from Abcam, E-cadherin and Vimentin from BD and total EGFR from Santa Cruz Biotechnology. All antibodies were used at a dilution of 1:1,000. Uncropped scans of the western blots from the main figures can be found in Supplementary Fig. 9.

### Generation of patient-derived resistant cell line models

The patient-derived cell line models MGH119, MGH121, MGH125, MGH126, MGH131-1, MGH131-2, MGH134 and MGH156 were developed on collagen-coated plates in ACL4 medium and transferred to RPMI. MGH157 was developed initially in RPMI. The cell line MGH141 was derived using the feeder system with irradiated fibroblasts (5,000 rad) from normal patient tissue. When a tumour cell majority was observed it was passaged off of the feeder layer and later transferred to RPMI medium for experiments. The development of a model was considered complete when it was independent of the feeder system, free of stromal cells and determined to maintain known patient tumour mutations. MGH119-R was derived *in vitro* from the treatment naive model, MGH119, through *in vitro* exposure to gefitinib, escalating from 10 nM to a final concentration of 1 μM.

### DNA extraction library construction and WES

Genomic DNA (gDNA) from normal liver, diaphragmatic tumour (NSCLC), lung tumour (SCLC) and liver tumour (SCLC) from patient #7 was extracted from OCT-embedded frozen tissue blocks using the DNAdvance kit from Agencourt. Three micrograms of gDNA from each sample were fragmented to approximately 150–200 bp by sonication and subjected to exome enrichment using the SureSelect^XT^ Human All Exon Target Enrichment system. Barcoded deep sequencing libraries for the exome-enriched gDNA fragments were constructed using Applied Biosystems SOLiD 5500 Fragment Library Core Kit. WES was performed with an Applied Biosystems SOLiD 5500 deep sequencer to generate paired-end colour space reads (50 nucleotides forward and 35 nucleotides reverse) by a multiplexed operation. The colour-space data were aligned to the human hg19 reference genome sequence by the Applied Biosystems LifeScope software to generate BAM files. Mutation calls were made using the muTect mutation calling software.

### Quantification of *RB1* gDNA levels by qPCR

*RB1* gene copy number was measured via a quantitative PCR assay that has been previously described^34^. Briefly, reaction samples containing 10 ng of gDNA with SYBR green master mix (Roche) were run on a LightCycler 480 (Roche) for quantification. Primer pairs amplifying exons 3 (F—5′- GAGCTACAGAAAAACATAGAAATCAGG -3′, R—5′- GGAAAATCCAGAATTCGTTTCC -3′), 13 (F—5′- GCATCTTTCCAGTTCGTATAAATACTC -3′, R—5′- CATAAAGTTACCCATAAATAGCAGCA -3′) and 25 (F—5′- ACAGCGACCGTGTGCTCAAA -3′, R—5′- AGCCAGGAGCAGTGCTGAGAC -3′) were used to obtain coverage of the beginning, middle and end of the *RB1* gene and primers amplifying long interspersed nuclear element-1 (LINE-1; F—5′- AAAGCCGCTCAACTACATGG -3′, R—5′- TGCTTTGAATGCGTCCCAGAG -3′) were used for each sample to serve as a loading control. A standard curve with normal female genomic DNA was generated for each primer pair in order to compare the tumour/cell line samples to a normal diploid sample.

### DNA extraction and array CGH analysis

DNA for the array CGH studies was extracted from formalin-fixed, paraffin-embedded tissues with the FormaPure kit from Agencourt. Agilent Sureprint G3 Cancer CGH+SNP 4 × 180 k Microarrays were used to identify genome-wide copy number alterations. Briefly, 1 μg of tumour and control DNA (normal female gDNA, Corriell Institute) were heated to 95 °C for 5 min. Random priming was used to label DNA with CY3-dUTP (control) and CY5-dUTP (tumour) dyes from the Agilent SureTag DNA Labeling kit. The labelled DNA was then purified over columns (Agilent) and mixed in equal proportion along with Cot-1 Human DNA (Agilent) for the hybridization steps. To hybridize the DNA to the array, incubation occurred first at 95 °C for 3 min for denaturation, followed by a 30-min pre-hybridization step at 37 °C and then a hybridization step for 35–40 h at 65 °C. Slides were then washed with Agilent Oligo ArrayCGH wash buffer 1 for 5 min at room temperature and wash buffer 2 for 1 min at 37 °C. Upon completion of the washes, slides were scanned using the G2505C Microarray Scanner (Agilent). The data were analysed using the Agilent CytoGenomics software v 2.0. CGH array data are available at GEO under accession number GSE64765 (super-series GSE64766).

### Immunohistochemistry

*RB*. The total RB IHC (Rabbit monoclonal Abcam #E182, 1:500) was performed on formalin-fixed, paraffin-embedded tissue sections using the Leica RX Bond Autostainer (Leica Biosystems). The sections cut 4–5 μm were baked off-line for 30 min in a 60 °C oven and then loaded onto the machine. The machine then de-waxed and hydrated online. Antigen retrieval was performed in ER 2 (Citrate buffer) for 20 min and stained using the Bond Polymer Refine Protocol under the IHC Modified F Protocol. Steps involved a 5-min Peroxide Block, a 15-min antibody/marker incubation, an 8-min post primary incubation, an 8-min polymer incubation, a 10-min DAB (diaminobenzidine) incubation and a 5-min haematoxylin incubation. Slides were then dehydrated, cleared, cover slipped and scored by a pathologist.

*EGFR*. IHC for EGFR was performed using EGFR D38B1 antibody (Cell Signaling #4267, 1:500 dilution in SignalStain Antibody Diluent) according to the manufacturer’s protocol. EGFR expression was evaluated using H score: 3 × percentage of tumour cells with high staining+2 × percentage of tumour cells with intermediate staining+1 × percentage of tumour cells with low staining, giving a range of 0–300. The expression in the normal bronchiolar epithelium was considered as a standard for a score of 2.

### Gene expression analysis

RNA from the MGH119, MGH119-R, MGH121, MGH125, MGH126, MGH134, MGH141, MGH157, MGH131-1 and MGH131-2 was isolated using the RNeasy kit (Qiagen). One microgram of RNA was submitted to the Dana Farber Cancer Institute Microarray Core Facility and was hybridized onto Affymetrix human U133plus DNA microarrays and raw expression data in the form of CEL files were obtained (ten samples). In parallel, CEL files for CCLE (http://www.broadinstitute.org/ccle/home) cell lines where the primary site was lung and the histology subtype 1 was non-small-cell carcinoma, squamous cell carcinoma, adenocarcinoma, large-cell carcinoma or small-cell carcinoma were collected (170 samples). Raw data from the Patient-derived cell lines (PDCL) and CCLE cell line CEL files were combined and normalized using RMA in the R Bioconductor package (PDCL data used in the analysis are available in GEO under accession GSE64322, super-series GSE64766). Hierarchical clustering in Supplementary Fig. 1 was performed using the 500 most differentially regulated genes in either the PDCL or CCLE samples. Probe sets were selected after removing low expressing and low variation probe sets with a simple variation filter where probe sets were thresholded to minimum values of 10 and then probe sets with less than fivefold variation between the minimum and maximum value or less than 50 absolute variation were removed (leaving 37,326–54,675 probe sets). After filtering, the 500 probe sets with largest standard deviation were selected using the 170 CCLE lung samples (upper half of Supplementary Fig. 1) or the 10 PDCL samples (lower half of Supplementary Fig. 1). Hierarchical clustering was performed on the log2 expression data for the combined data using Pearson correlation and the 500 CCLE-derived probe sets (upper half of Supplementary Fig. 1) or using Euclidean distance and the 500 PDCL-derived probe sets (lower half of Supplementary Fig. 1) in R using the heatmap.2 function with the complete agglomeration method.

### Methylation beadchip assay

Bisulfite-converted DNA was analysed using Illumina’s Infinium Human Methylation450 Beadchip Kit (WG-314-1001) according to the manufacturer’s instructions and data were acquired suing an Illumina iScan scanner. Raw.idat files were imported using the Bioconductor suite for R. Methylation levels, *β*, were represented according to the following equation:




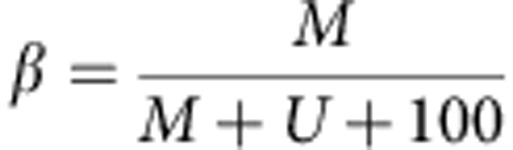




Where *M* represents the signal intensity of the methylated probe and *U* represents the signal intensity of the unmethylated probe. Probe dye bias was normalized using built-in control probes. Data points with a detection *P* value of <.01 were dropped. Finally, probes from X and Y chromosomes were excluded, leaving 473,864 unique probes. Principal component analysis was performed on quantile normalized data and informative probes with a standard deviation >0.2 were used for hierarchical clustering.

### miRNA expression analysis

Total RNA was prepared using the mirVana miRNA isolation kit (Invitrogen) per the manufacturer’s protocol. Specific Taqman assays for miRNAs 338 (002252), 101-3p (002253), 95-5p (000577), 106b-5p (000442), 17-5p (002308), 31 (001100), 21-5p (000397), 22-3p (000398), 29a-3p (002112), 29b-3p (000413) as well as RNU6B (001093) were also purchased from Invitrogen. These miRNAs represent the five most upregulated and downregulated in adenocarcinoma vs SCLC cell lines, respectively^17^. The relative expression of each miRNA was normalized with respect to RNU6B and then to the total RNA signal for each cell line as described previously^17^.

### Generation of shGFP/shRB and gefitinib-resistant cell lines

Viral constructs expressing shRNA targeting GFP and RB (targeting sequence—5′- GGTTGTGTCGAAATTGGATCA -3′) were obtained from Dr Nick Dyson and production and infection were completed as described previously^35^. Briefly, 293T cells were transfected with viral plasmids for shGFP or shRB along with VSV-G and delta8.91 using the TransIT-LT1 transfection reagent (Mirus). After 48 h, viral supernatant was collected and filtered. Infections were carried out with virus diluted 1 to 4 in media containing polybrene (8 μg ml^−1^). Following addition of virus, cells were spun at 1,200 revolutions per minute for 1 h. PC9 cells (2 μg ml^−1^) and HCC827 cells (1 μg ml^−1^) were selected in puromycin for 2 weeks. RB knockdown was confirmed by western blot analysis. Generation of gefitinib-resistant cells was carried out as described previously^36^. Briefly, cells were cultured in gefitinib-containing media starting at 10 nM and increased incrementally approximately every 2 weeks until the cells were able to freely replicate in 1 μM at roughly the same rate as parental cells (about 2 months). In addition, shGFP and shRB PC9 cells were made resistant to gefitinib by exposing parental cells to a high dose (300 nM) initially and then changing media and drug twice per week until resistant clones emerged and could be subcloned (6 weeks). In all cases, resistant cells were grown in the presence of gefitinib to maintain their resistant phenotype.

### Cultivating *in vivo* resistance in mouse xenograft models

Five million PC9 or HCC827 shGFP/shRB cells were mixed with Matrigel (BD Biosciences) in a 1:1 ratio and injected in both flanks of 48-week-old female athymic nude mice. Tumours took an average of 3 weeks to reach a size of 200 mm^3^ and then treatment was initiated. Gefitinib was delivered by oral gavage for 4 days on and 3 days off at a dose of 35 mg per kg. The PC9 tumours relapsed 4 months later while still on treatment. No detectable 827 tumours were visible after 4 months and treatments were discontinued. Following 6 weeks of drug holiday, the majority of 827 tumours had regrown and went back on treatment. In most cases, there was a moderate response followed by an eventual relapse. Experiments were approved by the Institutional Animal Care and Use Committee at Massachusetts General Hospital.

### Cell viability assays

Cell viability assays were carried out in a 96-well format with at least four replicates per condition. Cells were plated at a density of 2,000–4,000 cells per well depending on their respective size and growth rates: MGH125-2,000, MGH131-1-4,000, MGH131-2-4,000 and the rest at 3,000 cells per well. Following incubation with drug for the indicated concentration/time, CellTiter-Glo assay reagent (Promega) was added for 10 min and plates were read on a Centro LB960 microplate luminometer (Berthold Technologies).

## Additional information

**Accession codes**: Accession codes for data sets are as follows: microarray and array CGH are at GEO (GSE64322, GSE64765, super-series GSE64766) and WES is at European Genomics Association (EGAS00001001102).

**How to cite this article:** Niederst, M. J. *et al*. RB loss in resistant *EGFR* mutant lung adenocarcinomas that transform to small-cell lung cancer. *Nat. Commun.* 6:6377 doi: 10.1038/ncomms7377 (2015).

## Supplementary Material

Supplementary Information Supplementary Figures 1-9 and Supplementary Table 1

## Figures and Tables

**Table 1:** RB status of TKI-resistant patients.

**Patient**	**Cancer type**	**Resistance**	**Histology**	**RB status**	**Detection method**
1	Lung	Pre	Adeno	Pos	IHC
	Lung	Post	NE	Neg	IHC/genetic
	Lung	Post	NE	Neg	IHC/genetic
2	Lung	Pre	Adeno	Pos	IHC
	Lung	Pre	Adeno	Neg	IHC
	Lung	Post	NE	Neg	IHC
3	Lung	Pre	Adeno	Pos	IHC
	Lung	Post	NE	Neg	IHC
4	Lung	Post	NE	Neg	IHC
5	Lung	Post	NE	Neg	IHC
6	Lung	Pre	Adeno	Neg	IHC
	Lung	Post	NE	Neg	IHC/genetic*
7	Lung	Post	Adeno	Pos	IHC/genetic
	Lung	Post	NE	Neg	IHC/genetic
	Lung	Post	NE	Neg	Genetic
8	Lung	Post	Adeno	Pos	IHC
	Lung	Post	NE	Neg	IHC
9	Lung	Post	NE	Neg	IHC
10	Lung	Post	Adeno	Neg	IHC
11	Lung	Pre	Adeno	Pos	IHC
	Lung	Post	Adeno	Pos	IHC
12	Lung	Pre	Adeno	Pos	IHC
	Lung	Post	Adeno	Pos	IHC
13	Lung	Post	Adeno	Pos	IHC
14	Lung	Pre	Adeno	Pos	IHC
	Lung	Post	Adeno	Pos	IHC
15	Lung	Post	Adeno	Pos	IHC
16	Lung	Pre	Adeno	Pos	IHC
	Lung	Post	Adeno	Pos	IHC
17	Lung	Pre	Adeno	Pos	IHC
	Lung	Post	Adeno	Pos	IHC
18	Lung	Post	Adeno	Pos	IHC
19†	Lung	Intrinsic	NE	Neg	IHC

EGFR, epidermal growth factor receptor; IHC, immunohistochemistry; NE, neuroendocrine carcinoma; Neg, negative; Pos, positive; TKI, tyrosine kinase inhibitor.

RB status in pre/ post-TKI-resistant *EGFR* mutant lung cancers. EGFR TKI sensitivity, histology, RB expression and the detection method are listed for tumours from nine patients with resistant small-cell lung cancer (SCLC) and nine patients with resistant non-small-cell lung cancer. Patient 19 presented with classical SCLC with an *EGFR* mutation that was intrinsically resistant to EGFR TKI.

^*^Genetic data were from a cell line derived from that sample.

^†^Patient presented with *EGFR* mutant classical NE carcinoma and failed to respond to TKI.
