# Supplementary material for: RB loss in resistant EGFR mutant lung adenocarcinomas that transform to small-cell lung cancer
Source: Nat Commun. 2015 Mar 11;6:6377. doi: 10.1038/ncomms7377 (PMC4357281; doi:10.1038/ncomms7377)
Supplement: Supplementary Information — Supplementary Figures 1-9 and Supplementary Table 1 [file ncomms7377-s1.pdf]

1A

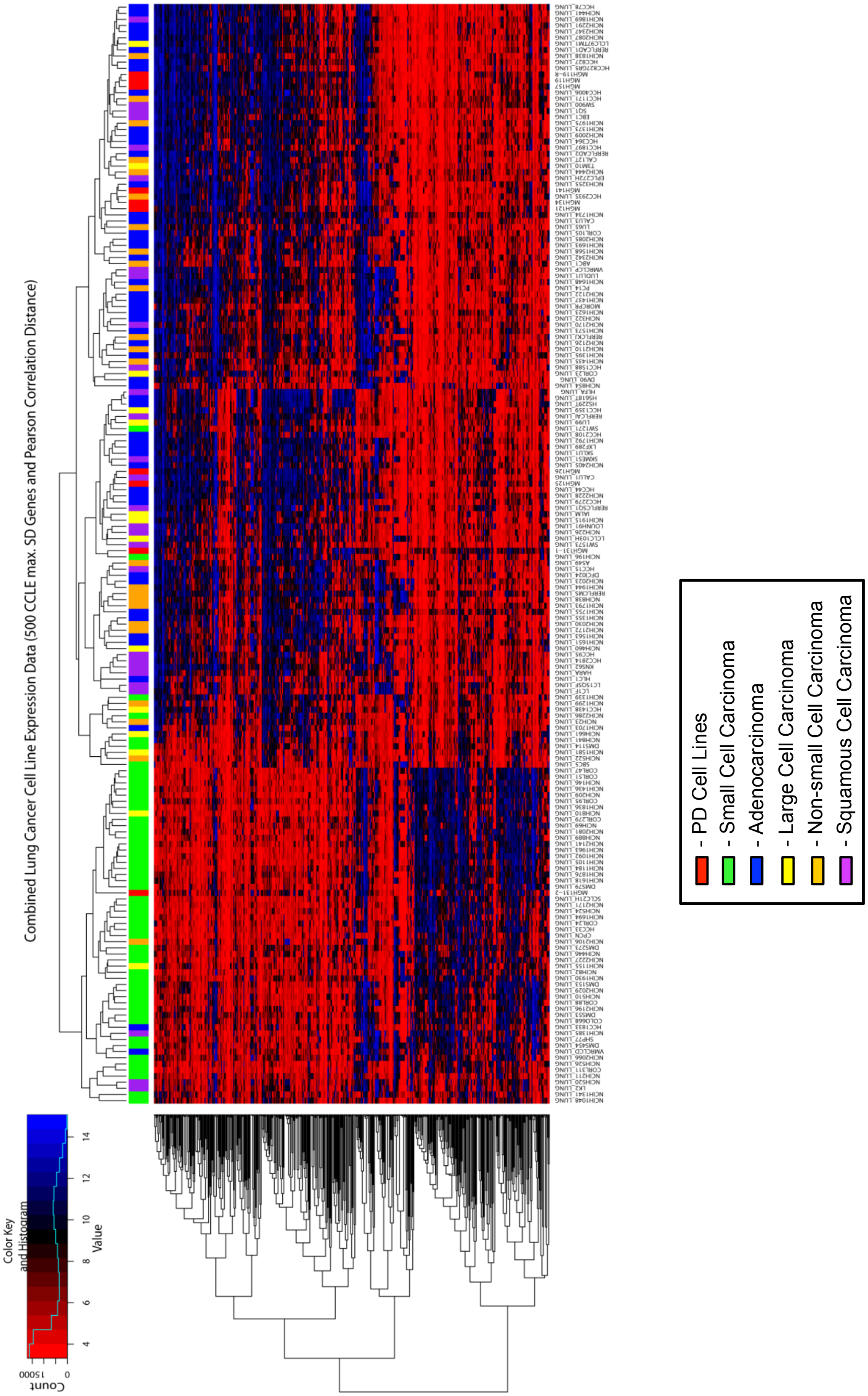

Combined Lung Cancer Cell Line Expression Data (500 CCLE max. SD Genes and Pearson Correlation Distance)
- PD Cell Lines
- Small Cell Carcinoma
- Adenocarcinoma
- Large Cell Carcinoma
- Non-small Cell Carcinoma
- Squamous Cell Carcinoma
Color Key and Histogram
Value
Count

1B

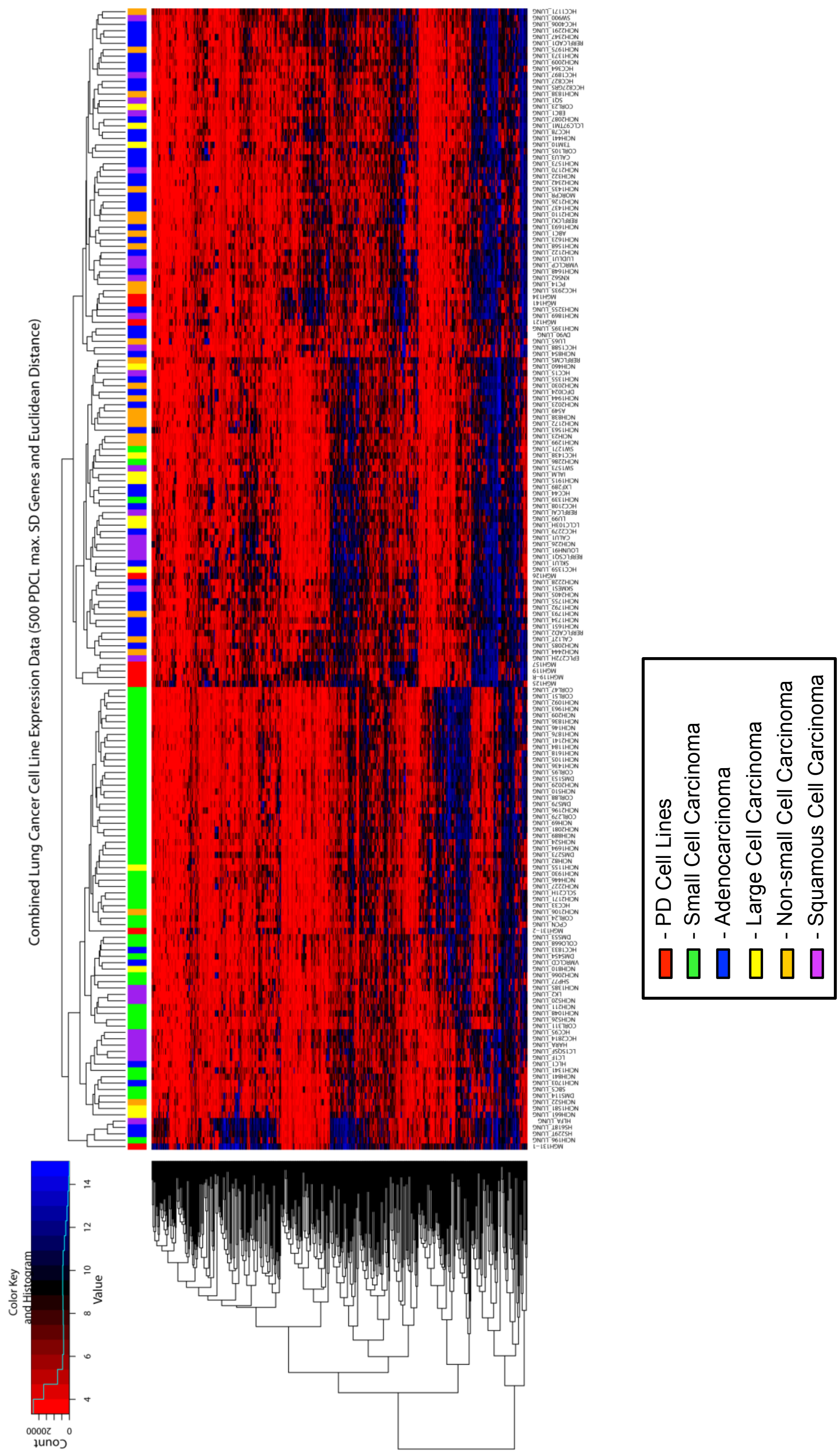

Combined Lung Cancer Cell Line Expression Data (500 PDCL max. SD Genes and Euclidean Distance)
Color Key and Histogram
Value
Count
- PD Cell Lines
- Small Cell Carcinoma
- Adenocarcinoma
- Large Cell Carcinoma
- Non-small Cell Carcinoma
- Squamous Cell Carcinoma

1C

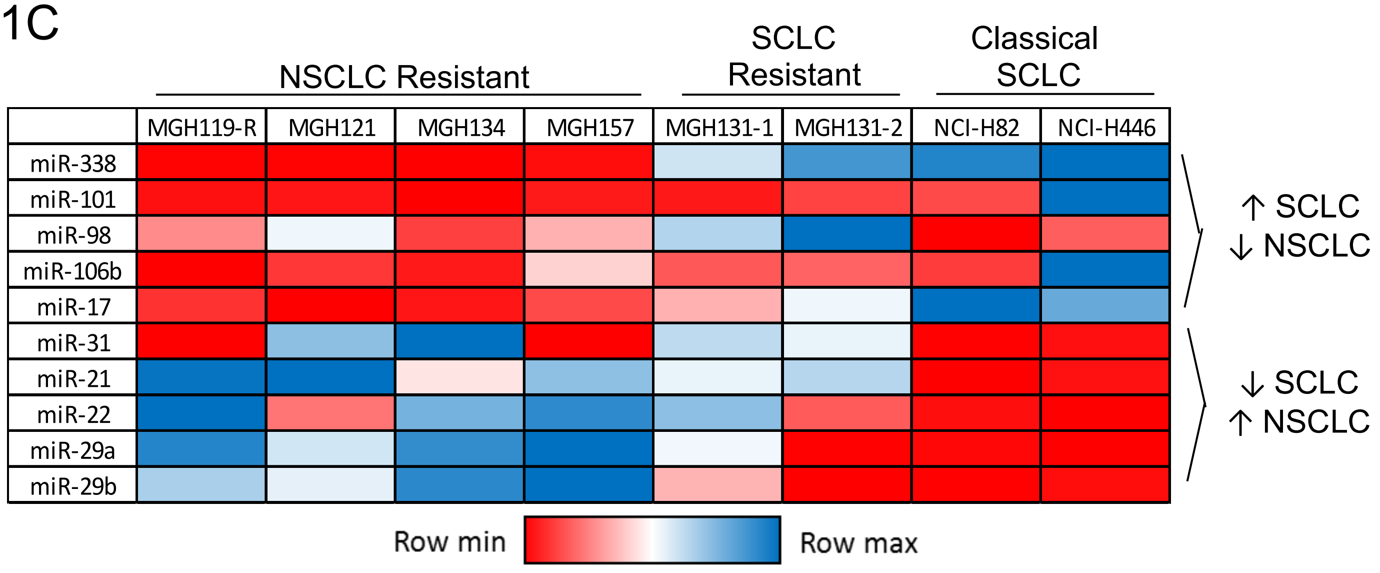

1D

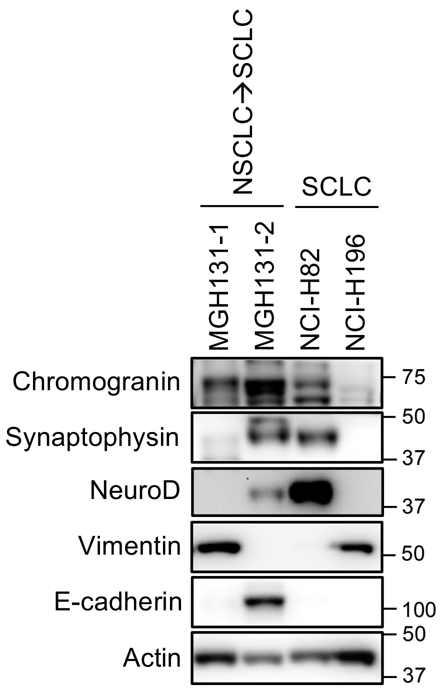

Supplementary Figure 1 – Gene expression of *EGFR* mutant SCLC resembles classical SCLC. A,B) Hierarchical clustering analysis of patient-derived resistant cell lines (see Fig. 1B) along with lung cancer cell lines from the Cancer Cell Line Encyclopedia (CCLE) with respect to the 500 genes with the highest variance among the CCLE lung cancer cell lines (A) or with respect to the 500 genes with highest variance among the patient derived cell lines (B). The color key shows the distribution of log2 expression levels with red representing low expression and blue representing high expression values. C) Taqman-based expression of miRNAs across a panel of cell lines derived from TKI resistant patients (See Fig. 1B). NCI-H82 and NCI-H446 are classical SCLC cell lines used as controls. Red indicates higher expression, blue indicates lower expression. The top and bottom 5 miRNAs represent those most differentially expressed between adenocarcinoma and SCLC, respectively[17]. D) Lysates from patient-derived *EGFR* mutant SCLC transformed as well as classical SCLC cell lines were probed with antibodies specific to chromogranin, synaptophysin, NeuroD, e-cadherin, vimentin and actin. These lysates were also probed in Fig. 2B.

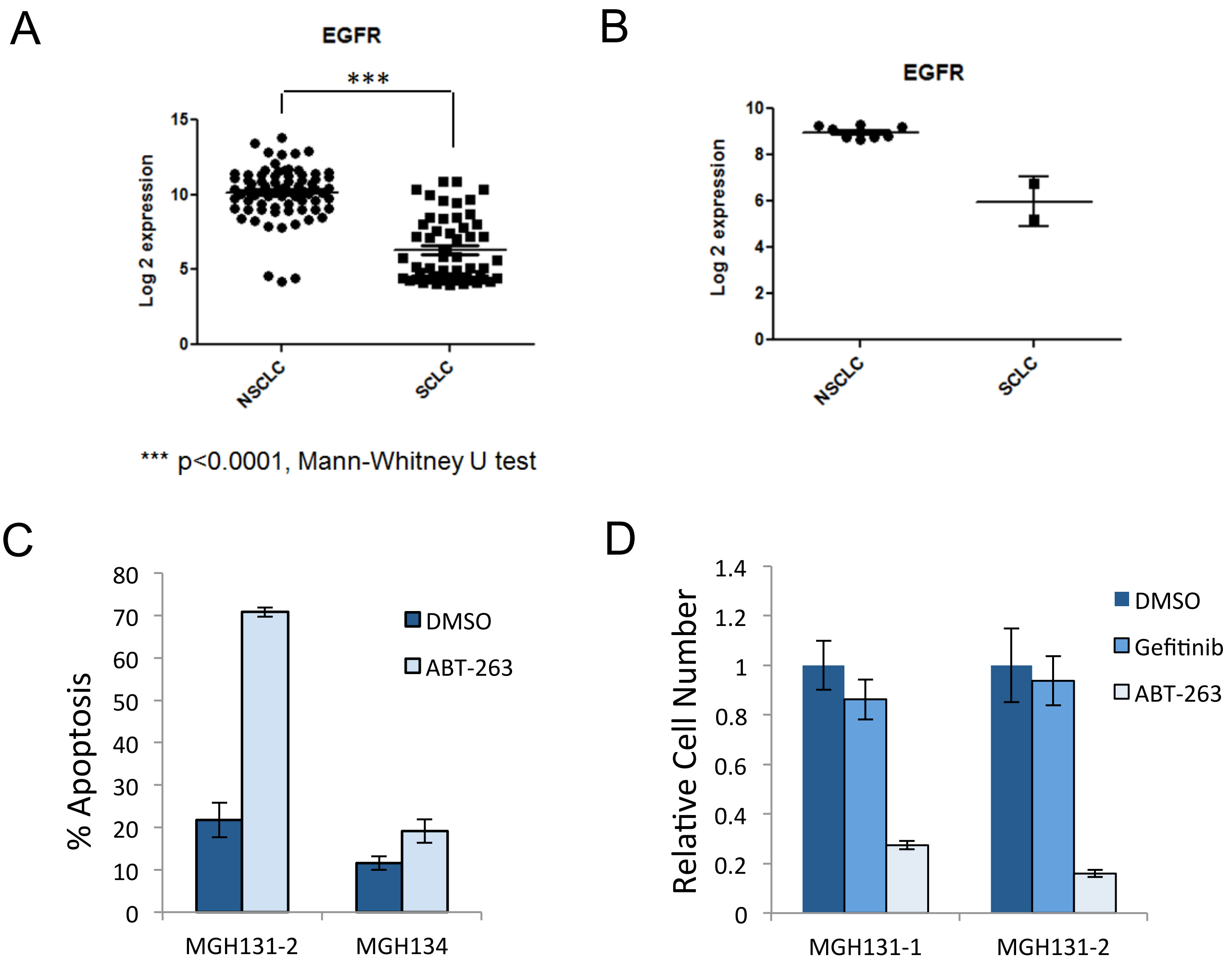

Supplementary Figure 2 – Resistant SCLC cells lose EGFR expression and are sensitive to ABT-263. A) EGFR expression values (Log2) from the CCLE database for NSCLC (n=75) and SCLC (n=54) cell lines[20]. *** p<0.0001, Mann-Whitney U test. B) EGFR expression values (Log2) from gene expression arrays for the TKI-resistant patient derived cell lines in Fig.1B. NSCLC n=8, SCLC n=2. C) MGH131-2 and MGH134 cells were treated with DMSO or 1μM ABT-263 for 48 hours. Apoptosis was measured by propidium iodide/Annexin V staining and flow cytometry. Assay was completed in triplicate and error bars represent standard error of the mean. D) Resistant *EGFR* mutant SCLC cell lines MGH131-1 and MGH131-2 were treated with DMSO, gefitinib (GEF, 1μM), ABT-263 (0.5μM) for three days. Cell viability was measured at day 3 in quadruplicate by the CellTiter-Glo assay. Error bars represent the standard error of the mean.

3A

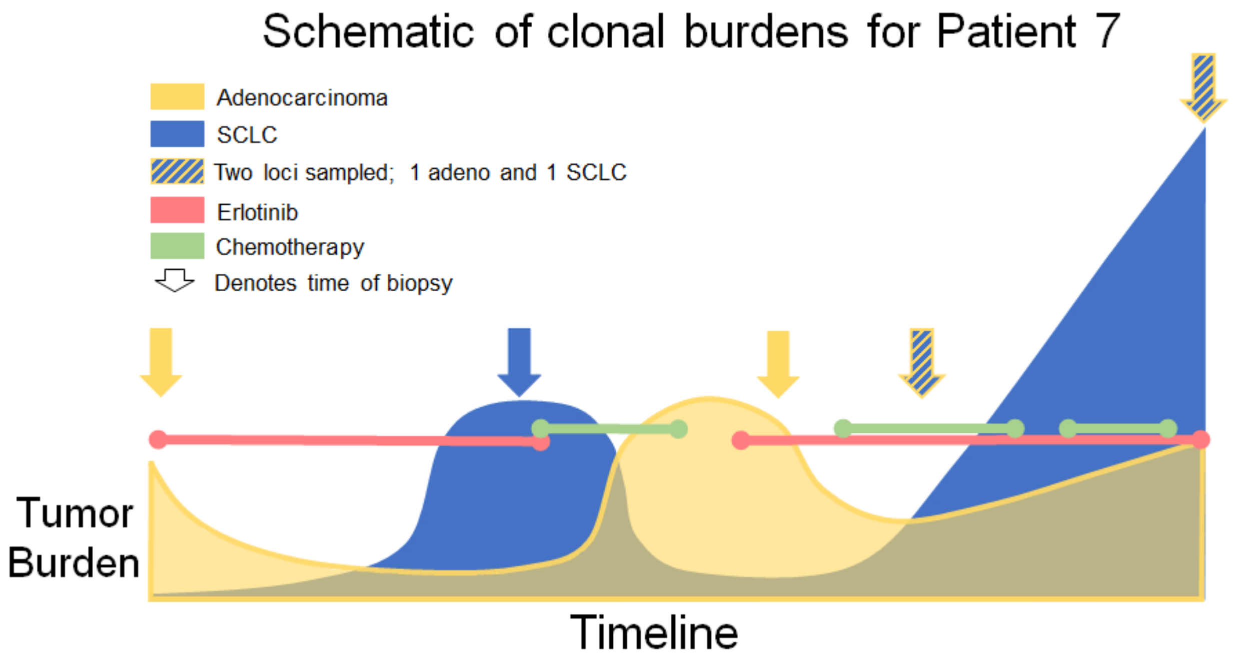
Schematic of clonal burdens for Patient 7
Adenocarcinoma
SCLC
Two loci sampled; 1 adeno and 1 SCLC
Erlotinib
Chemotherapy
Denotes time of biopsy
Tumor Burden
Timeline

3B

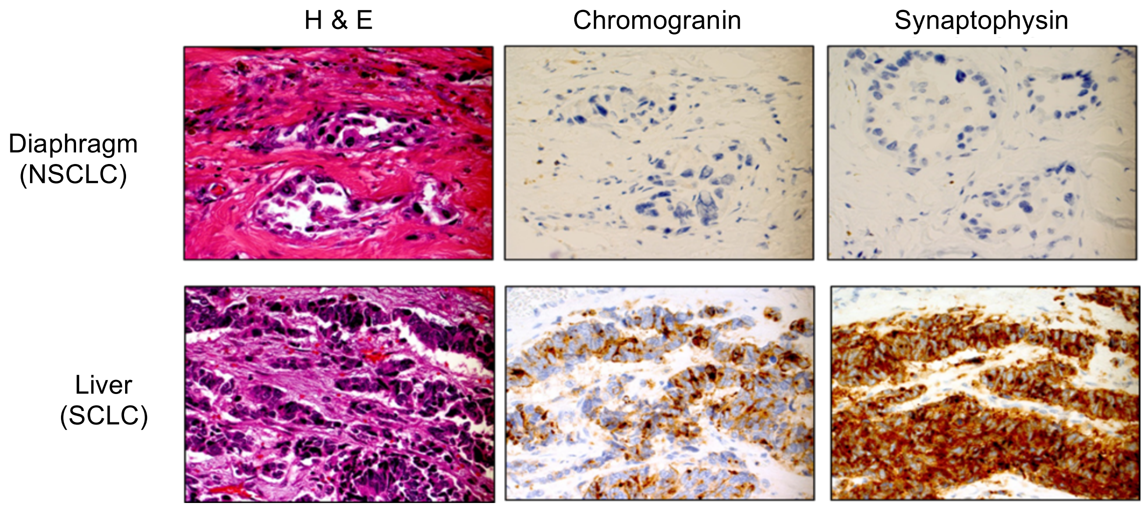
H & E
Chromogranin
Synaptophysin
Diaphragm (NSCLC)
Liver (SCLC)

3C

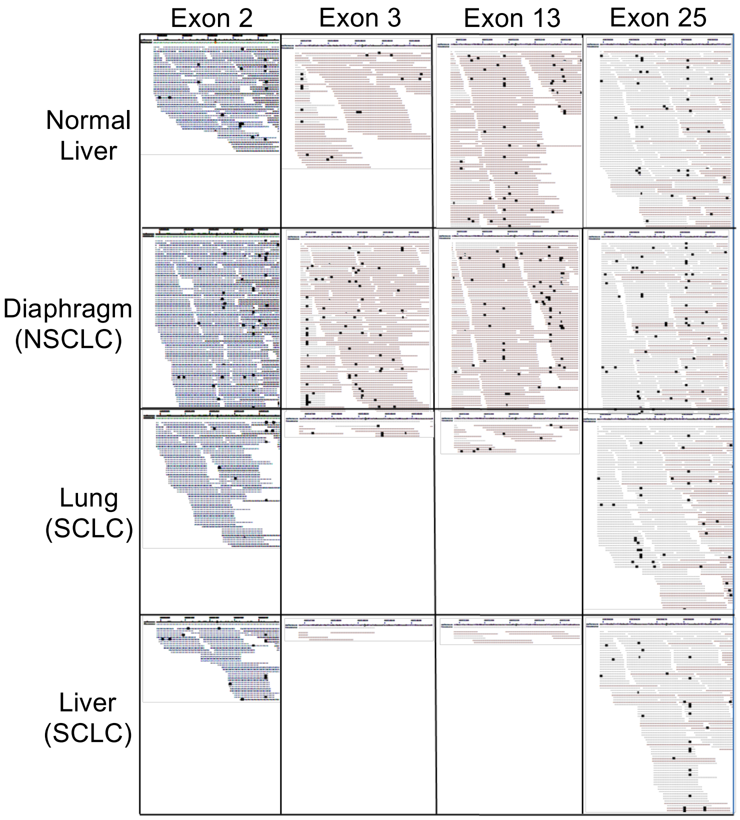

Supplementary Figure 3 – Resistant *EGFR* mutant SCLCs but not NSCLCs have loss of *RB1.* A) Model of the relative burden of adenocarcinoma and SCLC clones from Patient #7 over the course of treatment. B) *Left,* H & E staining of resistant tumors from the diaphragm (top) and liver (bottom). *Center and Right,* Immunohistochemistry for the neuroendocrine markers chromogranin and synaptophysin. C) Mapped sequencing reads from the exome sequencing data for exons 2, 3, 13, and 25 of the *RB1* locus from normal liver, diaphragm (NSCLC), lung (SCLC) and liver (SCLC).

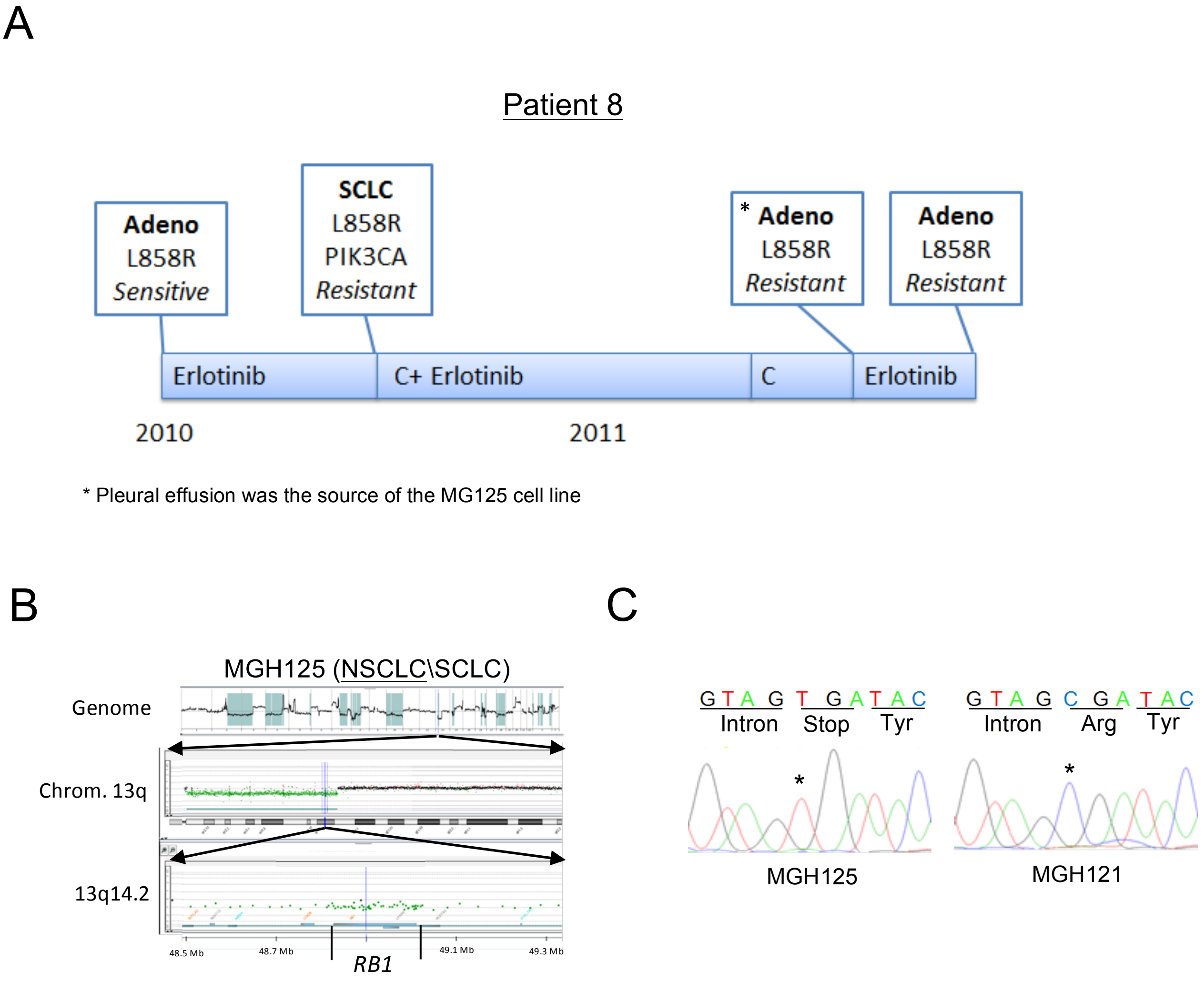

Supplementary Figure 4 – MGH125 (Patient #8) has 1 mutant and 1 deleted copy of RB. I. A) Longitudinal evaluation of the treatment and biopsy history of the patient (patient #8) whose resistant pleural effusion was the source of the MGH125 cell line. Text inside the bar indicates treatment regimen over time, text boxes above the bar each represent a biopsy/pleural effusion (or autopsy for final box). Bold - histology, regular text - genotype, italics – inferred sensitivity to EGFR TKI at time of biopsy/pleural effusion. C – Chemotherapy B) CGH array profile of the MGH125 cell line at level of the whole genome (top), chromosome 13q12.12-q32.2 (middle) and the 0.8Mb region flanking the *RB1* gene (Bottom). The *RB1* gene locus is depicted. C) Chromatogram indicating the R445* mutation in *RB1* from the MGH125 cells. The control MGH121 cells are a resistant *EGFR* mutant NSCLC line harboring T790M and positive for RB expression.

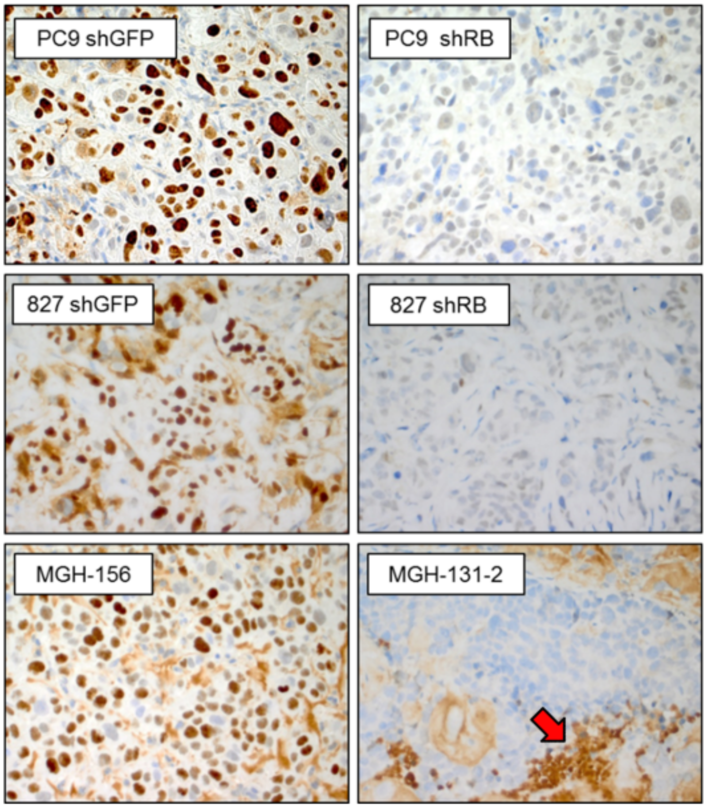

Supplementary Figure 5- RB IHC is both selective and sensitive. RB IHC staining of xenograft tumors of the indicated cell lines. Xenograft tumors were derived from PC9 and HCC827 cells with shRNA directed against GFP (control) or RB. MGH131-2 cells are derived from a resistant EGFR mutant SCLC and have bi-allelic *RB1* loss. Arrow indicates positive staining of pockets of stromal cells. MGH156 cells are resistant *EGFR* mutant NSCLC and express RB (Fig. 4C).

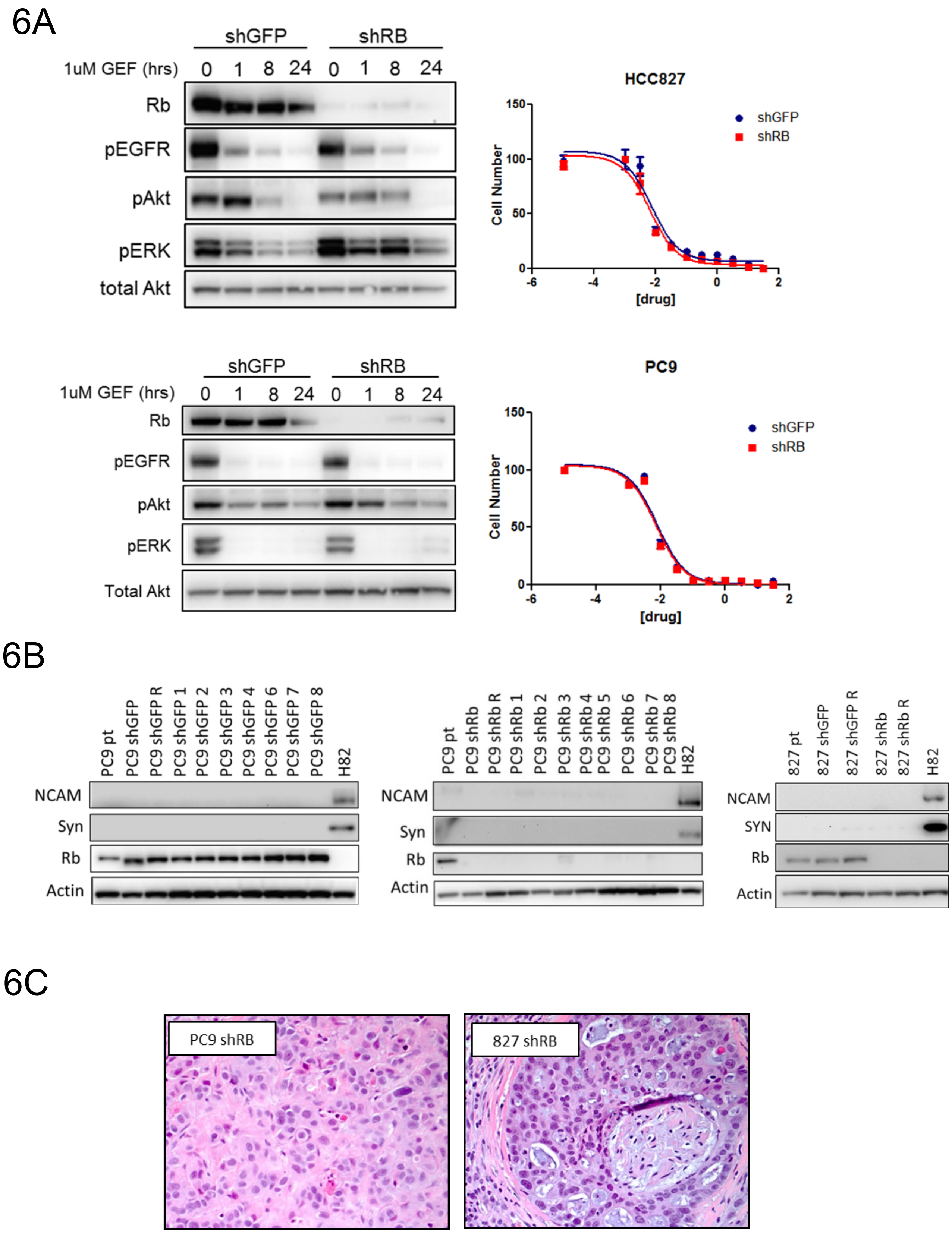

6A
shGFP
shRB
1uM GEF (hrs)
0 1 8 24 0 1 8 24
Rb
pEGFR
pAkt
pERK
total Akt
HCC827
shGFP
shRB
Cell Number
[drug]
PC9
Total Akt
6B
PC9 pt
PC9 shGFP
PC9 shGFP R
PC9 shGFP 1
PC9 shGFP 2
PC9 shGFP 3
PC9 shGFP 4
PC9 shGFP 6
PC9 shGFP 7
PC9 shGFP 8
H82
NCAM
Syn
Actin
PC9 shRb
PC9 shRb R
PC9 shRb 1
PC9 shRb 2
PC9 shRb 3
PC9 shRb 4
PC9 shRb 5
PC9 shRb 6
PC9 shRb 7
PC9 shRb 8
827 pt
827 shGFP
827 shGFP R
827 shRb
827 shRb R
SYN
6C
PC9 shRB
827 shRB

Supplementary Figure 6 – Depletion of RB does not promote resistance/transformation to SCLC. A) Left – *EGFR* mutant NSCLC cell lines HCC827 (top) and PC9 (bottom) stably infected with shRNA targeting GFP or RB were treated with 1μM GEF for 1, 8, or 24 hours. Lysates were probed with antibodies specific to pEGFR (Y1068), Rb, pAKT (308), AKT, and pERK (T202/Y204). Right – HCC827 shGFP and shRB (top) and PC9 shGFP and shRB (bottom) cells were treated with the indicated concentrations of gefitinib for 72 hours. Cell viability was measured by the CellTiter-Glo assay. Each data point was completed in sextuplicate and the error bars represent standard error of the mean. B) *Left and center panels,* Lysates from PC9 shGFP and shRB cells made resistant to gefinitib *in vitro* by incrementally increasing the dose of gefitinib (PC9 shGFP R and PC9 shRB R) or by exposure to a single dose of 300nM (PC9 shGFP and shRB clones 1-8) were probed with antibodies to the neuroendocrine (NE) markers NCAM and synaptophysin (Syn) as well as RB and Actin. Lysates from the SCLC cell line, H82, were used as a positive control for NE marker expression. *Right panel*, Lysates from HCC827 shGFP and shRB cells that were made resistant to gefitinib *in vitro* (827 shGFP R and 827 shRB R) were probed with antibodies to the neuroendocrine (NE) markers NCAM and synaptophysin as well as RB and Actin. C) H & E staining of PC9 shRB and 827 shRB cell xenograft tumors that were made resistant to gefitinib *in vivo*. The histology for these tumors is consistent with poorly differentiated adenocarcinoma.

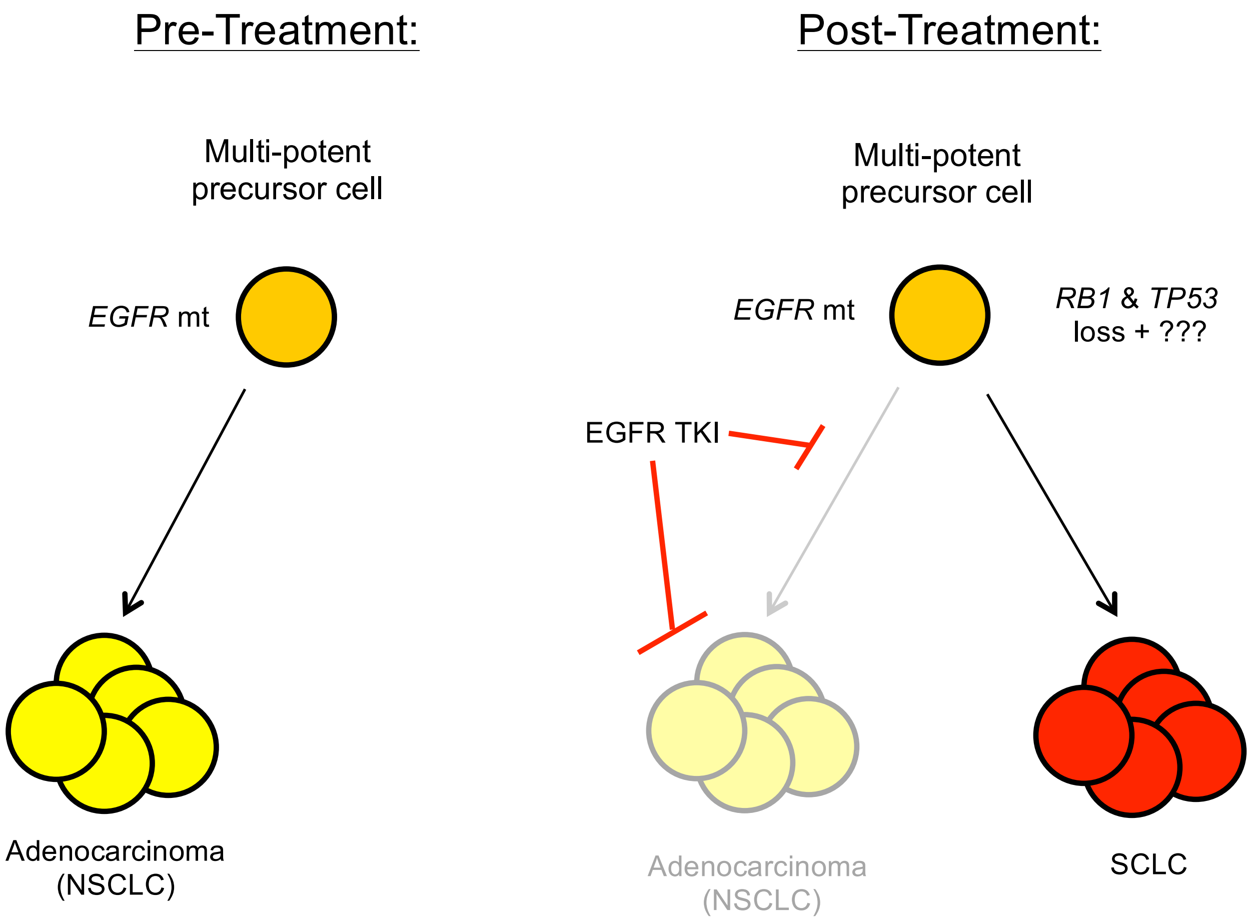

Supplementary Figure 7 – Putative model of NSCLC to SCLC transformation. In patients whose tumors transform from NSCLC to SCLC, the *EGFR* activating mutation occurs in a multi-potent cell that is capable of giving rise to both NSCLC and SCLC. *Left-* Prior to treatment, strong EGFR activation promotes differentiation along the adenocarcinoma lineage. *Right-* Following TKI treatment, the differentiated adenocarcinoma cells undergo apoptosis leaving undifferentiated multi-potent cells. Continued suppression of EGFR signaling prevents any further differentiation to NSCLC. Additional genetic/epigenetic changes such as *RB1* and *TP53* loss (and likely others) allow the precursor cells to escape along the SCLC lineage.

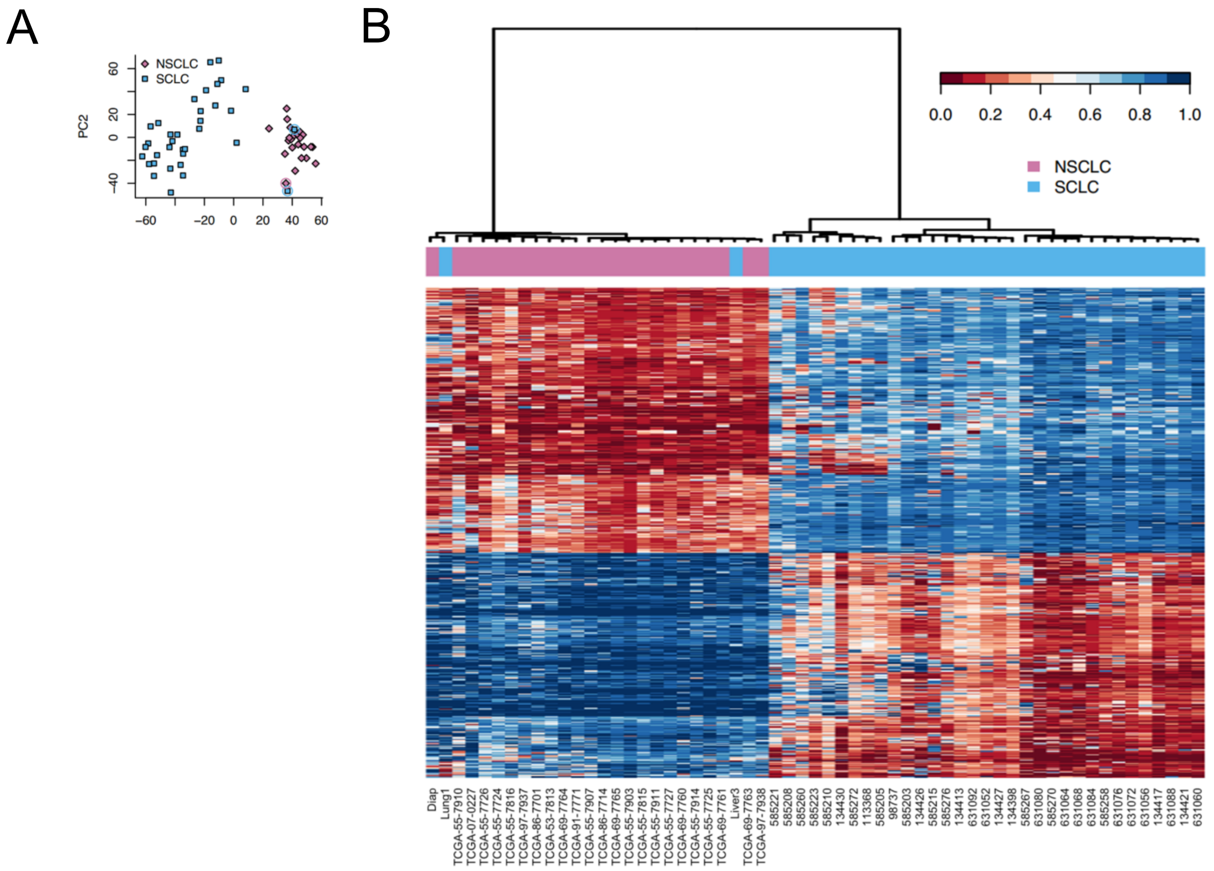

Supplementary Figure 8 – DNA methylation analysis of SCLC transformed cancers. Left - Principal component analysis of primary NSCLC samples (N=21) from The Cancer Genome Atlas (TCGA) lung adenocarcinoma project (LUAD) and primary SCLC samples (N=33) and three samples from Patient #7 representing both NSCLC and SCLC resistant tumors. Right - Unsupervised clustering of individual CpG sites based on differential methylation between primary NSCLC and SCLC samples (two-sided Wilcoxon rank sum test adjusted for false discovery rate by the method of Benjamini & Hochberg, shown with cutoffs of q <.01 and mean differential β >.5).

Figure 2B

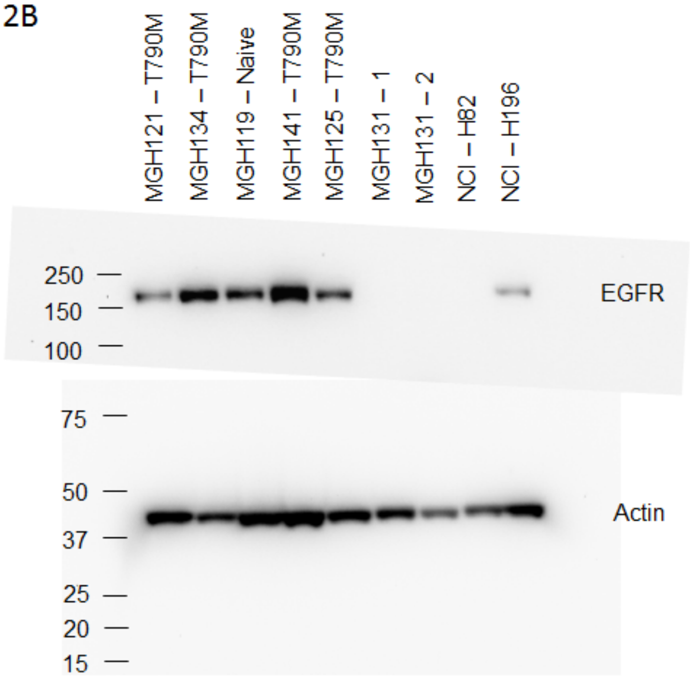

Figure 4C

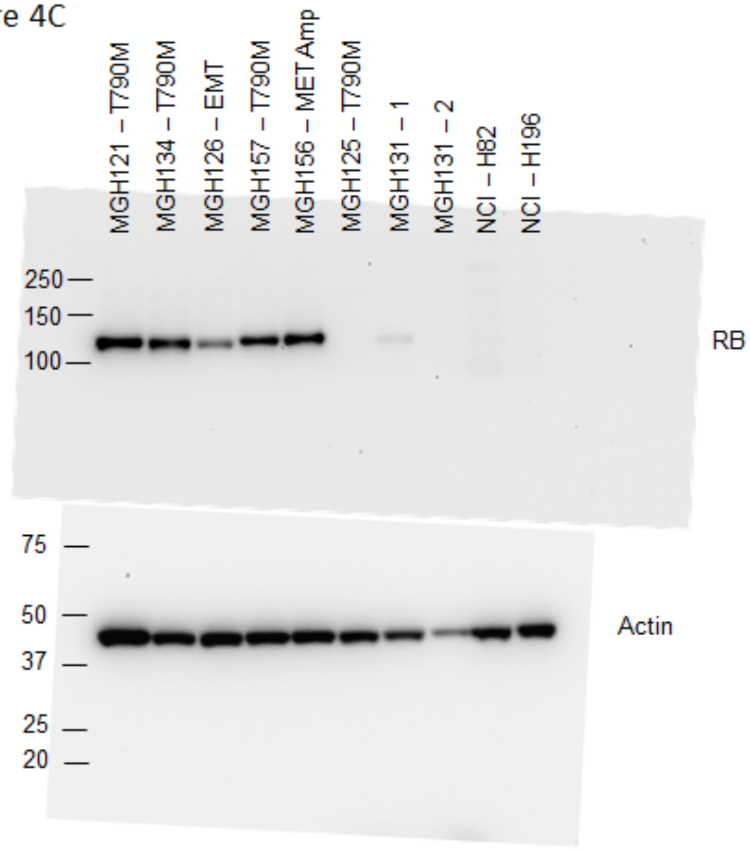

Supplementary Figure 9 – Uncropped blots from main figures.

ID #	Age	Sex	1° EGFR Mutation	Primary TKI	Start TKI	End TKI	Duration on TKI (mo.)	Resistance Mechanism(s)
1	52	F	Exon 19 Del	Gefitinib	9/1/2005	8/1/2007	23	SCLC Trans.
2	65	F	Exon 19 Del	Erlotinib	12/1/2010	8/1/2011	8	SCLC Trans.
3	69	F	Exon 19 Del	Erlotinib	8/1/2010	10/1/2012	26	SCLC Trans.
4	55	F	L858R	Erlotinib	4/1/2012	1/1/2013	9	SCLC Trans.
5	53	M	Exon 19 Del	Erlotinib	12/1/2010	7/26/2011	8	SCLC Trans, PIK3CA
6	43	M	Exon 19 Del	Erlotinib	8/3/2010	3/2/2011	7	SCLC Trans.
7	57	F	Exon 19 Del	Erlotinib	3/20/2008	5/13/2009	13	SCLC Trans, PIK3CA, T790M
8	64	F	L858R	Erlotinib	5/14/2010	10/6/2010	5	SCLC Trans, PIK3CA
9	55	F	Exon 19 Del	Erlotinib	7/1/2006	11/1/2009	40	SCLC Trans.
10	60	F	Exon 19 Del	Erlotinib	5/22/2009	12/22/2009	7	T790M
11	42	M	Exon 19 Del	Erlotinib	10/3/2008	2/24/2009	4	T790M, EGFR Amp
12	53	F	Exon 19 Del	Erlotinib	2/13/2009	9/20/2010	19	T790M
13	51	F	Exon 19 Del	Erlotinib	2/10/2009	8/4/2010	17	T790M
14	67	F	Exon 19 Del, G719x	Erlotinib	9/7/2010	5/17/2011	8	T790M
15	62	F	Exon 19 Del	Erlotinib	9/3/2008	5/3/2010	20	T790M
16	43	F	Exon 19 Del	Erlotinib	10/28/2010	5/19/2011	7	T790M
17	60	F	Exon 19 Del	Erlotinib	2/8/2010	7/12/2011	17	T790M
18	55	F	Exon 19 Del	Erlotinib	7/15/2010	10/4/2011	15	T790M, EGFR Amp
19*	73	M	Exon 19 Del	Erlotinib	7/16/2013	10/8/2013	No Response	N/A

*Patient presented with EGFR mutant classical SCLC and failed to respond to TKI.

Supplementary Table 1 - Details of the 19 patients whose cancers were included in the study.
